# Single‐Cell Dissection of Therapy‐Induced Remodeling Uncovers a Fibroblast‐Driven Immunosuppressive Niche and Targetable Vulnerabilities in Lethal Prostate Cancer

**DOI:** 10.1002/advs.202521088

**Published:** 2026-07-17

**Authors:** Yang Chen, Dandan Dong, Jinling Liao, Naikai Liao, Mengqi Liu, Qin Zhang, Siting Chen, Qi Liu, Ying Lu, Tianyu Li, Chunlin Zou, Qiong Song, Qiuyan Wang, Jian Liang, Qiji Xie, Chengyang Li, Mengyun Wang, Jiwen Cheng, Zengnan Mo, Gong‐Hong Wei

**Affiliations:** ^1^ Department of Urology The First Affiliated Hospital of Guangxi Medical University Nanning Guangxi China; ^2^ Center For Genomic and Personalized Medicine Guangxi Key Laboratory for Genomic and Personalized Medicine University Engineering Research Center of Digital Medicine and Healthcare, School of Life Science Guangxi Medical University Nanning Guangxi China; ^3^ Institute of Urology and Nephrology First Affiliated Hospital of Guangxi Medical University Guangxi Medical University Nanning Guangxi China; ^4^ Key Laboratory of Metabolism and Molecular Medicine of the Ministry of Education & Department of Biochemistry and Molecular Biology School of Basic Medical Sciences and Fudan University Shanghai Cancer Center Shanghai Medical College of Fudan University Shanghai China; ^5^ State Key Laboratory of Common Mechanism Research for Major Diseases Suzhou Institute of Systems Medicine Chinese Academy of Medical Sciences & Peking Union Medical College Suzhou Jiangsu China; ^6^ Disease Networks Research Unit Biocenter Oulu Faculty of Biochemistry and Molecular Medicine University of Oulu Oulu Finland; ^7^ Key Laboratory of Longevity and Aging‐Related Disease of Chinese Ministry of Education Center for Translational Medicine, School of Basic Medical Sciences Guangxi Medical University Nanning Guangxi China; ^8^ Cancer Institute Fudan University Shanghai Cancer Center Department of Oncology Shanghai Medical College of Fudan University Shanghai China

**Keywords:** androgen deprivation, complement, DPT^+^ fibroblast, neuroendocrine differentiation, NRXN1, T‐cell exhaustion, TSPAN1

## Abstract

Therapy resistance in prostate cancer arises from coordinated remodeling of malignant and stromal compartments, yet the mechanisms orchestrating this ecosystem adaptation remain elusive. Here, single‐cell RNA sequencing of longitudinal biopsies obtained before and after androgen‐deprivation therapy (ADT) delineated a therapy‐induced stromal lineage bifurcation toward APOD^+^ and DPT^+^ fibroblast states. DPT^+^ fibroblasts activated a C3‐ITGAX/ITGB2 complement signaling axis targeting macrophages, coinciding with suppression of M1 inflammatory programs, amplification of immune‐checkpoint signaling, and a shift of CD8^+^ T cells from cytotoxic to exhausted phenotypes. Concomitantly, we identified pre‐existing malignant epithelial subpopulations characterized by reduced AR/KLK3 activity and heightened chromosomal instability that preferentially persisted following therapy. Integrative multi‐omic analyses nominated TSPAN1 as a functional effector of castrate resistant prostate cancer (CRPC) and NRXN1 as a regulator of neuroendocrine plasticity through calcium‐dependent signaling programs. Genetic silencing of either gene suppressed proliferation, clonogenicity, migration, and tumor growth, while attenuating neuroendocrine features in vitro and in vivo. Spatial mapping, functional perturbation, and stromal‐epithelial co‐culture experiments mechanistically established a therapy‐induced DPT^+^ fibroblast‐complement circuit that enforced immune evasion and channels epithelial trajectories toward CRPC or neuroendocrine prostate cancer. Collectively, these findings defined the DPT^+^‐complement‐macrophage axis as an actionable vulnerability and position TSPAN1 and NRXN1 as therapeutic entry points to disrupt ADT‐driven tumor ecosystem remodeling in prostate cancer.

## Graphical abstract

Single‐cell longitudinal profiling reveals that androgen‐deprivation therapy induces a DPT^+^ fibroblast‐complement axis that suppresses macrophage inflammation and drives CD8^+^ T cell exhaustion in prostate cancer. Concurrently, resistant epithelial subpopulations persist and engage TSPAN1‐ and NRXN1‐mediated programs promoting CRPC and neuroendocrine plasticity, identifying actionable vulnerabilities within therapy‐remodeled tumor ecosystems.

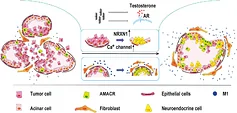

## 1 Introduction

Prostate cancer (PCa) remains one of the most prevalent malignancies in men worldwide [1], and androgen‐deprivation therapy (ADT) constitutes the cornerstone of systemic treatment for advanced disease [1, 2]. While ADT typically elicits substantial initial tumor regression, virtually all patients ultimately progress to castrate‐resistant prostate cancer (CRPC) or develop lineage‐plastic variants such as neuroendocrine prostate cancer (NEPC) [3, 4, 5]. This clinical trajectory reflects the dual impact of androgen deprivation. Beyond suppressing androgen receptor (AR) signaling, ADT profoundly reshapes the tumor microenvironment in ways that foster immune dysfunction and therapeutic resistance. Emerging evidence indicates that ADT rapidly remodels the tumor milieu, promoting the infiltration of regulatory immune populations and attenuating effector T‐cell activity [6]. Consistent with these observations, clinical trials combining ADT with immune checkpoint blockade have demonstrated limited efficacy [7], underscoring that therapy‐driven ecosystem remodeling represents a major obstacle to durable disease control.

Advances in single‐cell transcriptomics had begun to illuminate the diversity of epithelial and stromal compartments in PCa [8, 9, 10, 11, 12], revealing extensive malignant heterogeneity alongside distinct fibroblast states [13, 14]. Cancer‐associated fibroblasts (CAFs) are increasingly recognized as central orchestrators of therapy resistance through metabolic reprogramming, extracellular matrix remodeling, and paracrine signaling [15, 16]. Recent studies have further refined this framework by identifying specialized fibroblast subsets capable of actively modulating immune responses. Notably, iron‐loaded CAFs, termed FerroCAFs have been shown to promote immunosuppression in PCa through dysregulated iron metabolism and ferroptosis‐associated pathways [17]. Ferroptosis, a transition metal‐dependent form of regulated cell death driven by iron‐catalyzed lipid peroxidation, represents a mechanistic interface between metabolic stress and immune regulation [18]. These findings underscore that stromal compartments can shape immune landscapes through metal‐dependent biochemical programs. However, whether additional, mechanistically distinct fibroblast subsets arise dynamically under therapeutic pressure, and how such states coordinate stromal‐immune‐epithelial crosstalk, remains incompletely understood. Moreover, the extent to which systemic therapy reshapes fibroblast states to establish novel immunoregulatory niches has not been defined. Addressing these questions is essential for constructing an integrated framework that explains how treatment pressure orchestrates coordinated remodeling across the tumor ecosystem.

To interrogate therapy‐induced ecosystem dynamics, we performed longitudinal single‐cell RNA sequencing of paired human prostate cancer biopsies obtained before and after ADT, generating a high‐resolution atlas of the therapy‐exposed tumor ecosystem. Our analysis revealed a striking bifurcation of fibroblast states into APOD^+^ populations characterized by attenuated oncogenic signaling and DPT^+^ populations enriched for immunoregulatory programs. DPT^+^ fibroblasts secreted complement component C3, which engaged ITGAX/ITGB2 receptors on macrophages and promoted their polarization toward an immunosuppressive phenotype. Concurrently, CD8^+^ T cells exhibited a therapy‐associated transition from cytotoxic to exhausted states. Malignant epithelial cells displayed two distinct resistance trajectories: TSPAN1‐associated programs that enhanced proliferative and migratory capacities in CRPC‐like cells, and NRXN1‐driven neuroendocrine plasticity mediated through calcium signaling pathways. Collectively, these findings delineated a coordinated program of therapy‐induced remodeling that integrated stromal reprogramming, immune suppression, and epithelial adaptation.

Together, our results supported a unified model in which ADT induces a DPT^+^ CAF‐macrophage complement axis that reinforced immune escape and converges with epithelial persistence pathways to fuel therapy resistance. By linking fibroblast‐mediated immune modulation with epithelial lineage plasticity, this work advanced the conceptual understanding of treatment‐induced ecosystem remodeling beyond single‐compartment analyses. Importantly, it identified potential therapeutic vulnerabilities at multiple levels of the tumor ecosystem, including inhibition of complement signaling to restore antitumor immunity and disruption of TSPAN1‐ and NRXN1‐dependent epithelial programs to prevent CRPC and NEPC progression. These insights suggested combinatorial therapeutic strategies that addressed the systemic and microenvironmental consequences of androgen deprivation in prostate cancer.

## 2 Results

### 2.1 Construction of the Cellular Landscape From Benign Prostate to ADT‐Treated Prostate Cancer

To delineate cellular transitions underpinning PCa progression and therapeutic adaptation, we performed scRNA‐seq on 11 prostate tissue samples comprising five benign prostates, four untreated PCa specimens, and two PCa samples obtained after ADT. Following stringent quality control and removal of low‐quality and redundant cell populations (Figure S1), we generated a high‐confidence single‐cell atlas spanning benign, malignant, and therapy‐exposed states.

Unsupervised clustering revealed progressive expansion of cellular heterogeneity across disease stages. Whereas benign prostates contained nine major cell clusters, untreated PCa exhibited eleven clusters, and ADT‐treated tumors retained ten clusters (Figure 1a). This increased complexity was accompanied by dynamic remodeling of immune compartments within the tumor microenvironment. Specifically, T cells were markedly enriched in untreated PCa compared with benign tissues (6.21% vs. 20.45%) and remained elevated after ADT (15.1%), whereas macrophage populations progressively declined from benign prostate (8.75%) to untreated PCa (5.53%) and further following ADT (2.09%) (Figure 1b). Importantly, all identified clusters were consistently represented across individual patient samples (Figure 1c), indicating that the delineated cellular states are not driven by patient‐specific effects but instead represent robust and conserved transcriptional programs.

**FIGURE 1:**
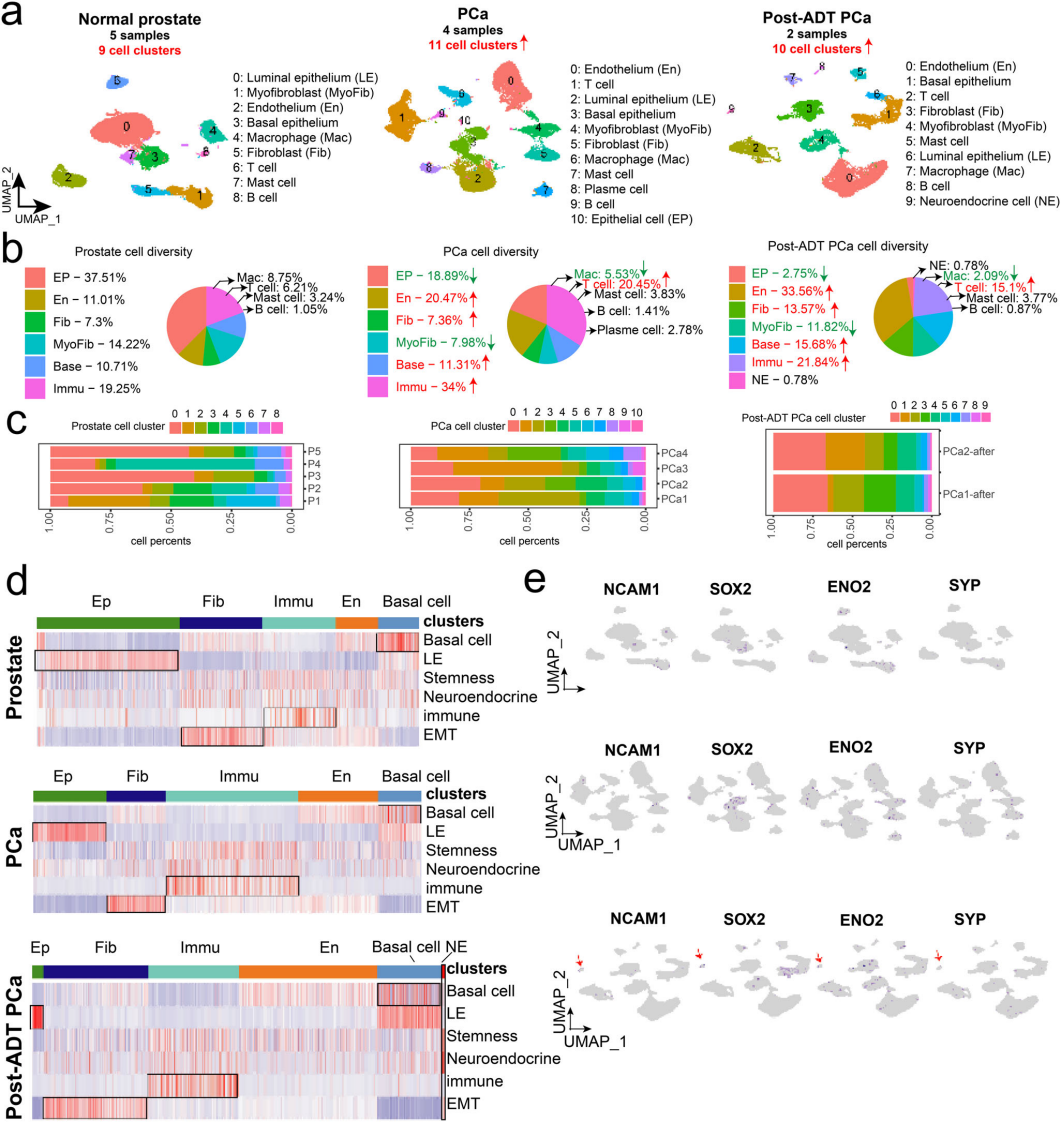
Single‐cell transcriptomic landscape reveals dynamic cellular remodeling from benign prostate to post‐ADT prostate cancer. (a) Uniform Manifold Approximation and Projection (UMAP) embedding of integrated single‐cell RNA sequencing profiles from benign prostates, treatment‐naïve prostate cancer (PCa), and ADT‐treated PCa specimens. Distinct cellular clusters are annotated to illustrate lineage identities and the progressive expansion of cellular heterogeneity across disease states. (b) Quantitative comparison of major cellular compartment proportions across benign prostate, untreated PCa, and ADT‐treated PCa. Immune remodeling is evident, including marked T‐cell enrichment in untreated PCa and persistent elevation following ADT, alongside progressive depletion of macrophage populations. Notably, epithelial populations decline sharply after ADT, accompanied by reciprocal expansion of endothelial cells. (c) Distribution of annotated cellular clusters across individual patients, demonstrating consistent representation of defined cell states and confirming robustness and reproducibility of the cellular atlas. (d) UMAP highlighting the emergence of a distinct neuroendocrine‐like cluster exclusively in ADT‐treated tumors, absent in benign and untreated PCa specimens, indicating therapy‐associated lineage reprogramming. (e) Feature expression plots showing canonical neuroendocrine markers (NCAM1, SOX2, ENO2, and SYP) selectively enriched in the ADT‐restricted NE cluster. Additional lineage‐associated gene signatures (basal, luminal epithelium, stemness, immune, and epithelial‐mesenchymal transition) further contextualize cellular state transitions and support adaptive neuroendocrine differentiation under androgen deprivation. Abbreviations: LE, luminal epithelium; MyoFib, myofibroblast; En, endothelial cell; Mac, macrophage; Fib, fibroblast; EP, epithelial cell; NE, neuroendocrine cell; Immu, immune cell; ADT, androgen‐deprivation therapy; EMT, epithelial‐mesenchymal transition.

Beyond immune remodeling, ADT elicited profound shifts in tissue composition, most notably a sharp depletion of epithelial (EP) cells accompanied by a reciprocal expansion of endothelial (En) populations (Figure 1a). These coordinated alterations indicated large‐scale remodeling of the tumor ecosystem under androgen‐deprived conditions.

Remarkably, ADT exposure was accompanied by the emergence of a previously unrecognized neuroendocrine‐like cellular cluster, which was absent in both benign and treatment‐naïve PCa samples (Figure 1d). This population expressed canonical neuroendocrine markers, including *NCAM1, SOX2, ENO2*, and *SYP* (Figure 1e), pointing to adaptive lineage reprogramming in response to androgen deprivation. The treatment‐restricted appearance of this cluster provides direct single‐cell evidence supporting adaptive lineage plasticity in response to androgen deprivation and offers mechanistic insight into the cellular origins of therapy‐induced neuroendocrine prostate cancer (NEPC), a clinically aggressive and treatment‐refractory subtype.

### 2.2 Single‐Cell Transcriptomic Profiling Before and After ADT

To minimize inter‐patient variability and directly capture therapy‐induced cellular dynamics, we next performed paired single‐cell RNA sequencing analysis on matched tumor specimens obtained from the same patients before and after ADT (Figures 2a and 3b). This paired design enabled high‐resolution delineation of lineage‐specific remodeling during therapeutic adaptation.

**FIGURE 2:**
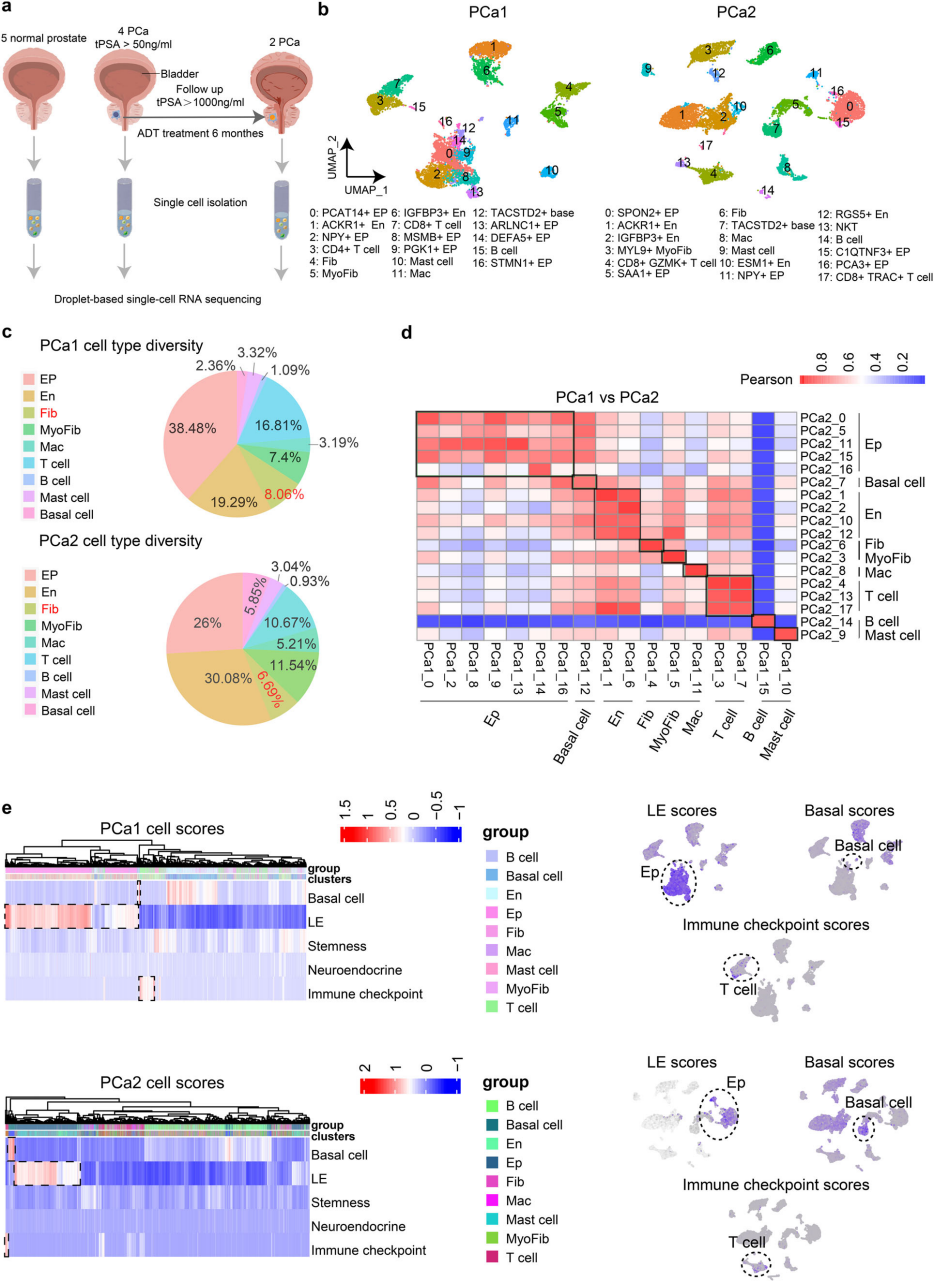
Paired single‐cell transcriptomic profiling delineates the hormone‐naïve cellular architecture of prostate cancer prior to androgen deprivation therapy. (a) Schematic overview of matched tumor sampling and single‐cell RNA sequencing. Fresh PCa tissues were obtained prior to ADT, dissociated into single‐cell suspensions, and subjected to high‐throughput scRNA‐seq to generate a high‐resolution cellular atlas of the hormone‐naïve tumor microenvironment. (b) UMAP embedding of 37 227 high‐quality single cells from two independent PCa specimens (PCa1 and PCa2) collected before ADT. Unsupervised clustering resolved transcriptionally distinct epithelial, stromal, immune, and endothelial compartments defined by canonical lineage markers, highlighting extensive inter‐ and intra‐tumoral heterogeneity. (c) Pie charts summarizing the relative abundance of major cell lineages in each tumor prior to treatment. (d) Pearson correlation analysis comparing transcriptional profiles of matched cell clusters between PCa1 and PCa2. Despite differences in cluster abundance, analogous lineages exhibited strong inter‐patient transcriptional concordance, indicating conserved lineage‐specific gene expression programs in untreated tumors. (e) Heatmap showing expression of canonical markers for basal epithelial cells, luminal epithelial cells, stemness‐associated genes, neuroendocrine differentiation markers, and immune checkpoint molecules across annotated clusters. Abbreviations: LE, luminal epithelium; MyoFib, myofibroblast; En: endothelium; Mac, macrophage; Fib: fibroblast, EP: epithelial cell; NE, neuroendocrine cell; Immu, immune cell; ADT, androgen‐deprivation therapy; NKT, natural killer T cell.

**FIGURE 3:**
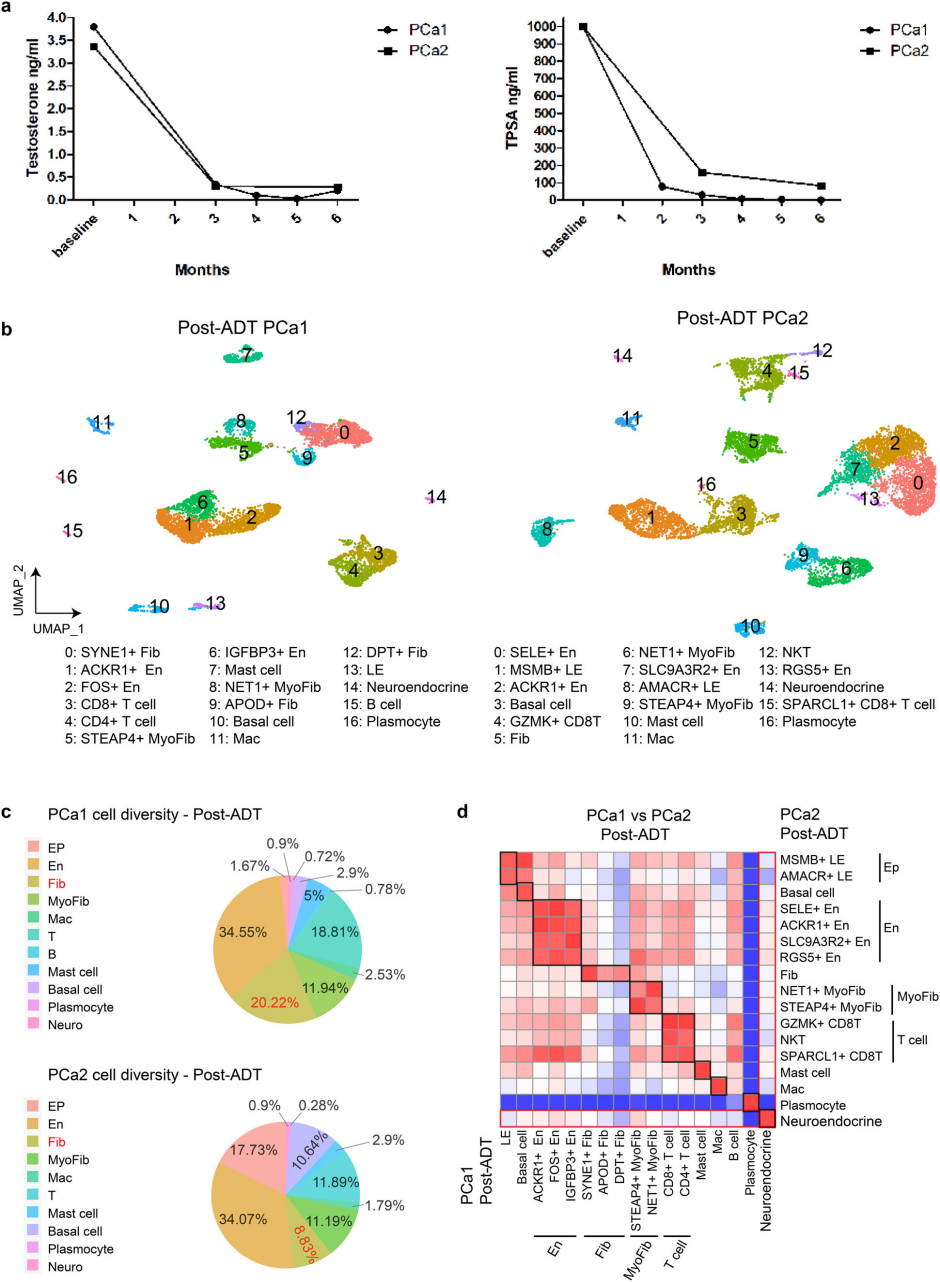
Androgen deprivation therapy remodels cellular composition and stromal architecture in prostate cancer. (a) Longitudinal serum measurements of testosterone and prostate‐specific antigen (PSA) in two patients undergoing six months of ADT, demonstrating systemic therapeutic response. (b) UMAP visualization of single‐cell transcriptomic profiles from matched post‐ADT PCa tissues, revealing the remodeled epithelial, stromal, endothelial, and immune compartments. (c) Quantitative comparison of cell‐type proportions after ADT, highlighting epithelial contraction accompanied by expansion of endothelial and fibroblast populations. (d) Pearson correlation analysis of transcriptionally defined cell clusters across the two post‐ADT tumors, illustrating conserved lineage‐specific programs despite inter‐patient variability in proportional shifts. Abbreviations: LE, luminal epithelium; EP, epithelial cell; NE, neuroendocrine cell; Fib, fibroblast; MyoFib, myofibroblast; En, endothelium; Mac, macrophage; Immu, immune cell; NKT, natural killer T cell; ADT, androgen‐deprivation therapy.

Prior to treatment, unsupervised clustering identified 17 (PCa1) and 18 (PCa2) transcriptionally distinct cell clusters in two independent PCa samples, respectively (Figure 2b). These clusters encompassed diverse epithelial, stromal, immune, and endothelial compartments, each defined by canonical lineage markers. Luminal epithelial cells were characterized by KRT8 and KRT18 expression, whereas basal epithelial cells expressed KRT5 and KRT19. Fibroblast populations included LUM/DCN/CFD‐positive fibroblasts and TAGLN/MYH11/ACTG2‐positive myofibroblasts. Immune subsets comprised C1QA/C1QB/C1QC‐positive macrophages, CD79A/CD79B‐positive B cells, and CD69/CD3D/CD52‐positive T cells. Endothelial populations displayed distinct subtype signatures, including ACKR1+, IGFBP3+, and ESM1+ endothelial cells marked by EMCN, CHD5, and ACKR1 expression (Figure 2b; Figure S2). Collectively, this high‐resolution mapping underscores the remarkable cellular heterogeneity of the hormone‐naïve PCa microenvironment.

Although epithelial and endothelial compartments predominated in both tumors before and after therapy, ADT induced striking and directionally coordinated shifts in their relative proportions. In PCa1, epithelial cells decreased dramatically from 38.48% to 1.67%, whereas endothelial cells expanded from 19.29% to 34.55%. In PCa2, epithelial cells declined from 26.00% to 17.73%, accompanied by an increase in endothelial cells from 30.08% to 34.07% (Figures 2c and 3c). Thus, despite quantitative differences between patients, both tumors exhibited epithelial contraction coupled with endothelial expansion following androgen deprivation, suggesting conserved remodeling of tissue architecture under hormonal stress.

To further resolve epithelial diversity prior to treatment, we subclustered the naïve epithelial compartment. In PCa1, eight transcriptionally distinct epithelial subpopulations were identified (PCAT14+ EP, NPY+ EP, MSMB+ EP, PGK1+ EP, ARLNC1+ EP, DEFA5+ EP, STMN1+ EP, and basal cell), whereas PCa2 harbored six epithelial subclusters (SPON2+ EP, NPY+ EP, SAA1+ EP, C1QTNF3+ EP, PCA3+ EP, and basal cell) (Figure 2b). This extensive intratumoral heterogeneity likely reflects functional diversification relevant to therapeutic responsiveness and disease trajectory. Strikingly, after ADT, only two to three epithelial clusters persisted in each tumor (Figure 3b), suggesting strong selective pressure and the potential survival of residual cell states capable of seeding recurrence.

Despite evident inter‐patient variability in cluster composition and proportional shifts, conserved lineage‐specific transcriptional programs were maintained across analogous cell types before and after treatment (Figures 2d and 3d). Luminal and basal epithelial populations consistently expressed canonical lineage markers, validating cluster annotation and identity (Figure 2e). Moreover, T‐cell clusters displayed enriched expression of immune checkpoint molecules in the hormone‐naïve setting, highlighting a potentially immunoreactive microenvironment prior to endocrine intervention. Importantly, neuroendocrine markers were absent in all pre‐treatment samples (Figure 2e), indicating that neuroendocrine‐like states do not represent a pre‐existing minor population but instead arise as a therapy‐induced adaptive program (Figures 1d,e, 2e, and 3d).

Together, these paired single‐cell analyses established a dynamic atlas of PCa remodeling during androgen deprivation. ADT drives profound reorganization of both tumor and stromal compartments, reshaping epithelial diversity, vascular architecture, and immune states. These findings illuminated the cellular trajectories underlying neoadjuvant treatment responses and provided mechanistic insight into early evolutionary events that may predispose tumors toward castration resistance.

### 2.3 ADT Induces Fibroblast Differentiation and Reprograms the Tumor Microenvironment

Clinically, both patients exhibited robust biochemical and pathological responses following six months of ADT. Serum testosterone levels declined to castrate range (PCa1: 0.20 ng/mL; PCa2: 0.28 ng/mL), accompanied by marked reductions in total PSA (PCa1: 0.85 ng/mL; PCa2: 82.62 ng/mL; Figure 3a). These systemic responses were paralleled by histopathological remission (Table S1), confirming effective tumor suppression and establishing a clinical framework for investigating therapy‐induced cellular remodeling.

Building on these observations, single‐cell transcriptomic profiling revealed profound restructuring of the tumor microenvironment. Among stromal compartments, fibroblast expansion emerged as a consistent hallmark of therapeutic adaptation. This increase was particularly pronounced in PCa1, which exhibited a greater PSA decline (8.1% to 20.2%), and was also evident in PCa2 (6.7% to 8.8%; Figures 2b,c and 3b,c). The coordinated enrichment of fibroblasts across patients suggests that stromal plasticity represents a conserved response to androgen deprivation rather than a patient‐specific phenomenon.

To resolve this stromal heterogeneity, fibroblasts from PCa1 were further partitioned into three transcriptionally distinct subsets sharing a common lineage identity (Figure 3b,d; Figure S2). Although global transcriptional profiles remained highly concordant before and after treatment (R^2^ > 0.9), subset‐specific divergence was evident. APOD^+^ and DPT^+^ fibroblasts showed the greatest deviation following ADT (R^2^ = 0.36 and 0.78, respectively), with APOD^+^ fibroblasts adopting the most distinct transcriptional state (Figure 4a). Pseudotime trajectory analysis positioned APOD^+^ and DPT^+^ fibroblasts along terminal differentiation branches (Figure 4b), consistent with therapy‐driven lineage progression. The presence of three fibroblast subsets and two myofibroblast populations was further validated by immunofluorescence staining of representative marker genes (Figure 4c). Across all three fibroblasts, androgen receptor (AR) expression was broadly suppressed after treatment, indicating lineage‐wide adaptation to androgen deprivation.

**FIGURE 4:**
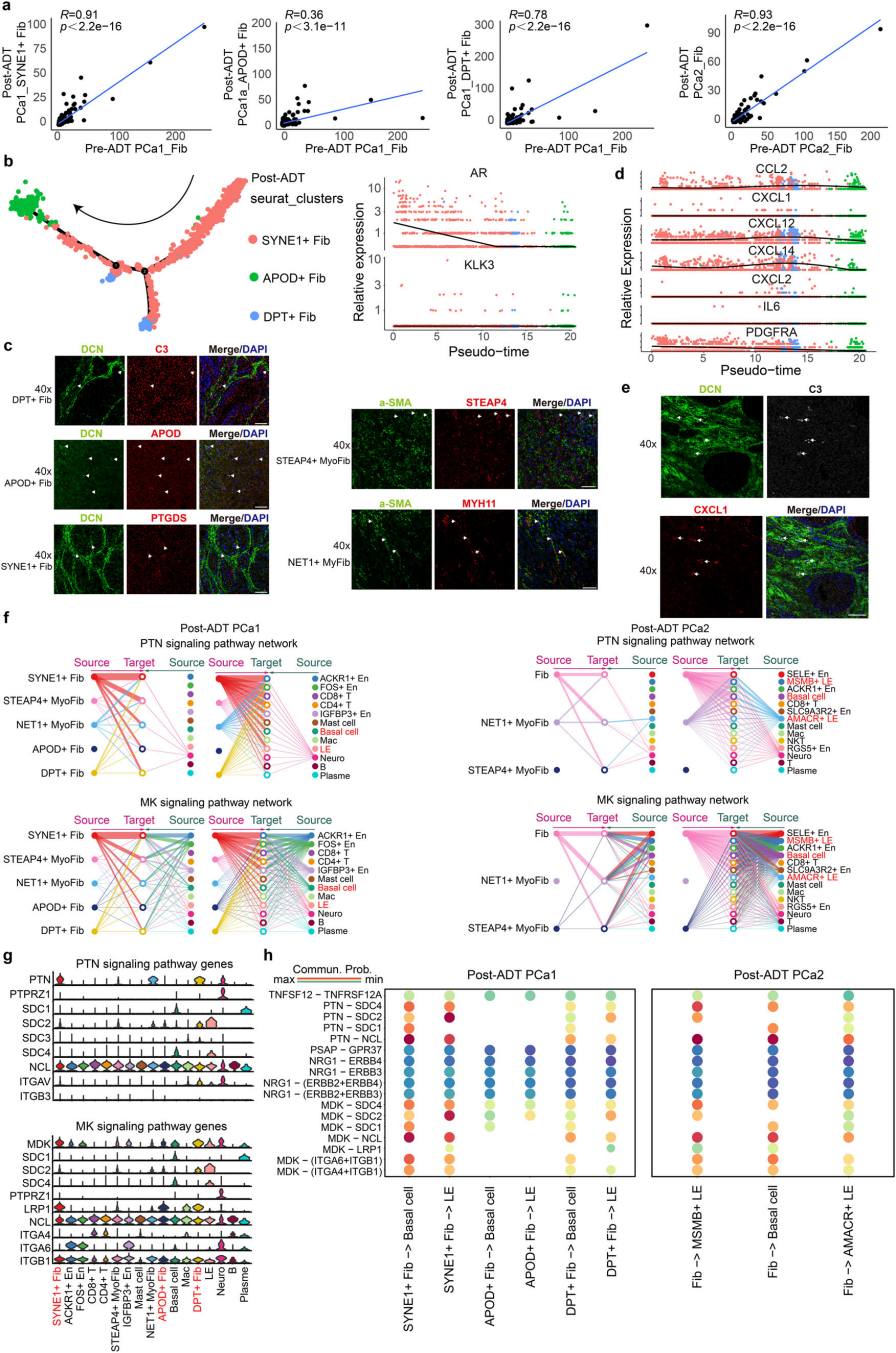
ADT promotes fibroblast differentiation and attenuates stromal inflammatory and oncogenic signaling. (a) Transcriptional correlation of fibroblast subsets before and after ADT, highlighting subset‐specific divergence, with APOD^+^ fibroblast exhibiting the greatest treatment‐induced shift. (b) Pseudotime trajectory analysis in PCa1 delineating lineage progression toward terminally differentiated APOD^+^ and DPT^+^ states following therapy. (c) Immunofluorescence validation of three fibroblast subsets and two myofibroblast populations using representative marker genes, confirming post‐treatment stromal heterogeneity. (d) Expression dynamics of inflammatory chemokines (CCL2, CXCL1, CXCL12, CXCL14) and stromal markers along with pseudotime, demonstrating progressive attenuation of pro‐inflammatory programs in differentiated subsets. (e) Immunofluorescence showing predominant CXCL1 co‐expression in DPT^+^ fibroblasts, supporting their relatively higher inflammatory signaling state. (f) Cell–cell communication analysis highlighting conserved PTN and MK pathways interactions across PCa samples. (g) Subset‐resolved signaling activity revealing reduced PTN and MK pathway engagement in differentiated fibroblasts, with complete PTN pathway inactivation in APOD^+^ cells. (h) Ligand‐receptor interaction mapping between fibroblasts and epithelial compartments, demonstrating diminished PTN–NCL and MDK–NCL signaling from APOD^+^ fibroblasts, consistent with reduced epithelial support.

Functional interrogation revealed that fibroblast heterogeneity was tightly linked to differential inflammatory programs. Compared with DPT^+^ fibroblasts, APOD^+^ and SYNE1^+^ subsets expressed markedly lower levels of chemokines including *CCL2, CXCL1, CXCL12*, and *CXCL14* (Figure 4d; Figure S3a), indicating a transition toward a less inflammatory stromal phenotype. Immunofluorescence confirmed *CXCL1* co‐expression predominantly in DPT^+^ fibroblasts (Figure 4e). These findings suggest that ADT promotes differentiation toward stromal states with reduced pro‐inflammatory and potentially less tumor‐supportive signaling capacity.

Consistent with this interpretation, intercellular communication analysis identified fibroblasts as central signaling hubs in the post‐treatment niche. Together with endothelial cells, fibroblasts maintained conserved ligand‐receptor interactions involving PTN, MK, TWEAK, PSAP, and NRG pathways (Figure 4f; Figure S3b–d), mediating epithelial‐stromal crosstalk through autocrine and paracrine circuits. Notably, signaling intensity was attenuated in APOD^+^ and DPT^+^ subsets, with APOD^+^ fibroblasts exhibiting complete loss of PTN pathway activity (Figure 4g; Figure S4). Ligand‐receptor mapping further identified MDK‐NCL and PTN‐NCL interactions as dominant fibroblast‐epithelial axes; however, reduced PTN expression rendered APOD^+^ fibroblasts largely incapable of effective communication with basal or luminal epithelial populations (Figure 4h). These features defined APOD^+^ fibroblasts as a differentiated, low‐signaling stromal state associated with diminished oncogenic support.

Collectively, these results demonstrated that ADT not only suppressed epithelial tumor compartments but also profoundly reprogrammed the stromal microenvironment by promoting fibroblast differentiation into less inflammatory and less communicative states. By delineating fibroblast lineage plasticity, inflammatory remodeling, and altered intercellular signaling networks, this study identified stromal remodeling as a central and previously underappreciated mechanism of therapeutic adaptation in prostate cancer.

### 2.4 DPT^+^ Fibroblasts Orchestrate Macrophage Polarization Toward an Immunosuppressive State

Gene ontology (GO) analysis of the DPT^+^ fibroblast cluster revealed a striking enrichment of immune‐regulatory processes (Figure S3a), suggesting that this population functions as a critical immunomodulatory component within the tumor microenvironment. To further define their role in intercellular communication, we performed comprehensive cell‐cell interaction analyses across the tumor microenvironment. These analyses revealed that DPT^+^ fibroblasts actively engage in multiple signaling networks, including complement, ANGPTL, GH, and SEMA3 pathways (Figure 5a and Figure S5a), highlighting their broad signaling capacity and potential to influence diverse cellular compartments within the tumor ecosystem.

**FIGURE 5:**
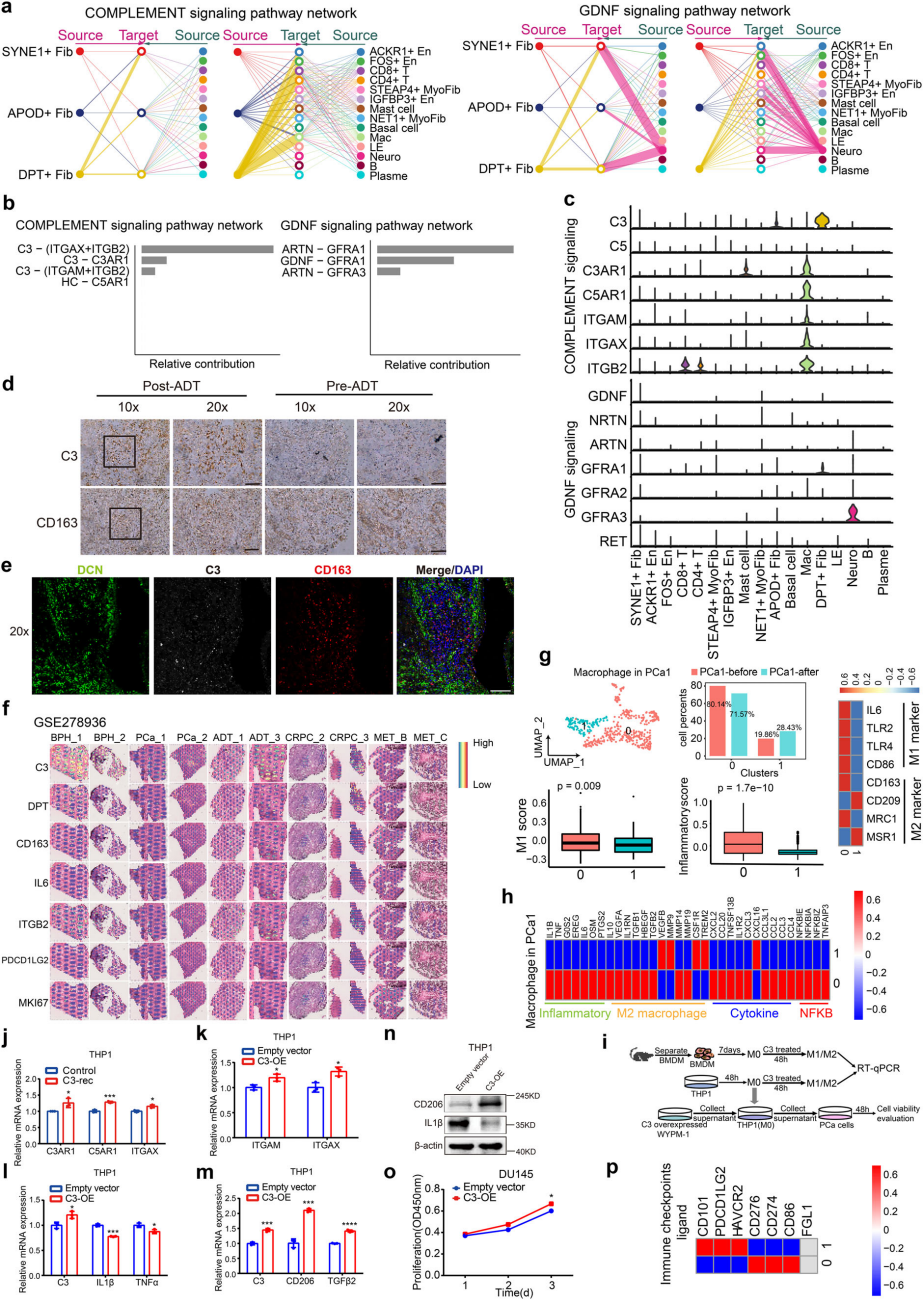
DPT^+^ fibroblasts suppress M1 macrophage activity and promote M2 polarization through complement signaling. (a) Cell–cell communication analysis revealing activation of the complement and GDNF signaling pathways in DPT^+^ fibroblasts, illustrating both outgoing (ligand‐mediated) and incoming (receptor‐mediated) interactions within the tumor microenvironment. (b) Quantitative contribution of individual ligand‐receptor pairs to complement and GDNF pathway activity, identifying C3‐centered signaling as a dominant mediator of DPT^+^ fibroblast–macrophage communication. (c) Single‐sample gene expression profiling of complement and GDNF pathway components in the first PCa specimen, demonstrating pathways upregulation in DPT^+^ fibroblast‐enriched regions. (d, e) Immunohistochemistry (d) and immunofluorescence (e) analyses showing increased macrophage accumulation surrounding C3‐expressing fibroblasts following ADT, indicating spatial immune remodeling. (f) Spatial transcriptomic analysis confirming enhanced co‐localized expression of C3, DPT, CD163, and PDCD1LG2 after ADT, supporting coordinated activation of fibroblast‐driven immunosuppressive programs. (g) Re‐clustering of macrophages before and after ADT identified two transcriptionally distinct macrophage populations and revealed treatment‐associated compositional shifts. (h) Differential gene expression analysis of inflammatory markers, M2‐associated genes, cytokines, and NF‐κB signaling components in the two macrophage subsets, demonstrating suppression of pro‐inflammatory programs and enrichment of alternative activation signatures. (i,o) Functional assays demonstrating that C3 (C3‐rec) directly promoted C3 receptors and M2 macrophages gene expression. Up‐regulated C3 (C3‐OE) in fibroblasts induced M2 macrophage polarization and PCa cell proliferation markedly. (p) Expression profiling of immune checkpoint ligands in macrophages, highlighting upregulation of immunosuppressive molecules and suggesting a DPT^+^ fibroblast‐driven mechanism of immune evasion following ADT.

Notably, DPT^+^ fibroblasts also emerged as prominent downstream responders of GDNF signaling, with neuroendocrine tumor cells serving as the principal ligand source (Figure 5a). This previously underappreciated fibroblast‐neuroendocrine crosstalk added an additional layer of regulatory complexity to PCa biology and positioned DPT^+^ fibroblasts as key integrative hubs within the tumor microenvironmental signaling hierarchy. Consistently, signaling activity in DPT^+^ fibroblasts was further elevated in neuroendocrine and metastatic PCa, as well as following ADT (Figure S5b), supporting a context‐dependent and castration‐associated expansion of their tumor‐promoting functions. Upstream regulatory analysis further revealed activation of FOXP1 and FOXP2 (Figure S5c), implicating lineage‐associated transcriptional reprogramming as a potential mechanistic driver of this phenotypic transition.

Among the enriched pathways, complement signaling emerged as particularly dominant. Specifically, the C3‐(ITGAX^+^ITGB2) ligand‐receptor axis displayed strong activity between DPT^+^ fibroblasts and macrophages (Figure 5b). Concordantly, macrophages exhibited robust expression of the complement receptors ITGAX and ITGB2 (Figure 5c), implicating complement‐mediated communication as a principal mechanism through which DPT^+^ fibroblasts influence macrophage behavior.

To determine the functional consequence of this interaction, we conducted integrative analyses across both bulk and single‐cell transcriptomic datasets. These analyses revealed a positive correlation between C3 expression and M2 macrophage‐associated signaling signatures (Figure S6a–d). Notably, ADT significantly increased C3 expression, accompanied by enhanced M2 macrophage signaling activity. In contrast, C3 levels exhibited an inverse correlation with M1‐associated inflammatory signatures, indicating a relative shift toward an immunosuppressive, M2‐dominant macrophage landscape following ADT (Figure S6a–d).

We next sought to spatially and histologically validate the predicted interaction between complement‐producing fibroblasts and macrophages in PCa. Immunostaining analyses of clinical PCa specimens confirmed the presence of C3‐expressing fibroblasts and revealed a pronounced enrichment of CD163^+^ M2‐like macrophages in close proximity to C3‐enriched stromal regions following ADT (Figure 5d,e). Consistently, spatial transcriptomic profiling demonstrated elevated C3 expression in post‐ADT tissues compared with pre‐treatment samples, accompanied by upregulation of DPT, CD163, and PDCD1LG2 (Figure 5f), collectively reinforcing the spatial and functional coupling between complement‐producing fibroblasts and immunosuppressive macrophages. Notably, these findings implicate ADT as a key driver of stromal‐immune reprogramming within the tumor microenvironment.

To determine the functional consequences of this interaction, we next profiled macrophage states before and after hormone therapy. Unsupervised clustering identified two transcriptionally distinct macrophage subsets: Cluster 0, which was predominant in pre‐treatment samples but decreased after therapy, and Cluster 1, which was enriched after treatment, increasing from 19.86% pre‐ADT to 28.43% post‐ADT (Figure 5g). Cluster 0 displayed stronger M1 macrophage signaling and a more pro‐inflammatory phenotype (Figure 5g). In contrast, post‐treatment macrophages exhibited increased expression of M2‐associated genes and reduced expression of pro‐inflammatory and cytokine‐related genes (Figure 5h), consistent with suppression of classical M1‐like inflammatory programs. Mechanistically, in vitro assays showed that C3 exposure promoted macrophage polarization from an M1‐like toward an M2‐like phenotype, accompanied by upregulation of complement receptors, including C3AR1, C5AR1, ITGAM, and ITGAX (Figure 5j; Figure S6e–i). Moreover, enforced C3 upregulation in fibroblasts directly induced M2 polarization and further enhanced PCa cell proliferation in co‐culture systems (Figure 5i,k–o). Together, these findings support a model in which DPT^+^^+^ fibroblast‐derived complement signaling suppresses macrophage inflammatory activity while reinforcing alternative activation states.

Concomitantly, post‐treatment macrophages displayed enhanced expression of immune checkpoint molecules, most notably PDCD1LG2 (Figure 5p). This coordinated transcriptional program, characterized by attenuated inflammatory capacity alongside heightened checkpoint expression, indicates that macrophages are reprogrammed toward an immunoregulatory state that both dampens anti‐tumor immunity and facilitates immune evasion under ADT.

Collectively, our findings demonstrate that DPT^+^ fibroblasts orchestrate macrophage polarization toward an immunosuppressive phenotype through complement‐dependent signaling. By simultaneously suppressing pro‐inflammatory responses and amplifying immune checkpoint pathways, this fibroblast‐macrophage axis establishes a microenvironment conducive to immune escape in the context of ADT.

### 2.5 ADT Induces CD8^+^ T Cell Exhaustion

As central mediators of antitumor immunity, T cells emerged as critical components of the complement‐regulated network orchestrated by DPT^+^ fibroblasts (Figure 5c). In the first PCa case, T cells segregated into CD8^+^ and CD4^+^ subpopulations, both of which modestly expanded following ADT. Notably, CD4^+^ T cells, previously implicated in promoting tumor progression [19], were entirely absent in the second patient (Figures 2b and 3b), underscoring marked inter‐patient heterogeneity in immune composition. Importantly, even prior to treatment, both cases exhibited baseline activation of immune checkpoint pathways (Figure 2e), indicating that immunosuppressive signaling was already established in the pretreatment microenvironment.

To delineate functional state transitions, we performed a high‐resolution analysis of CD8^+^ T cells. ADT induced the emergence of three distinct clusters, in which C0 displayed co‐expression of naïve‐associated markers (CCR7, TCF7, IL7R) alongside exhaustion‐associated transcription factors (EOMES, TOX, NFATC2) [17], suggesting a transitional or early dysfunctional phenotype. Clusters C1 and C3 were uniquely characterized by high expression of HSPA1A and HSPA1B, defining a stress‐responsive CD8^+^ T cell population (TSTR) previously linked to immunotherapy resistance [19]. In contrast, before therapy, CD8^+^ T cells were predominantly represented by the C2 cluster, which exhibited a classical cytotoxic phenotype marked by robust expression of effector molecules, including GZMB, GZMH, and GNLY (Figure 6a,b) [20]. Although exhaustion markers such as HAVCR2 were detectable at baseline, they coexisted with immune‐activating signals (Figure 6c), consistent with a functionally competent cytotoxic T cell compartment prior to hormonal intervention.

**FIGURE 6:**
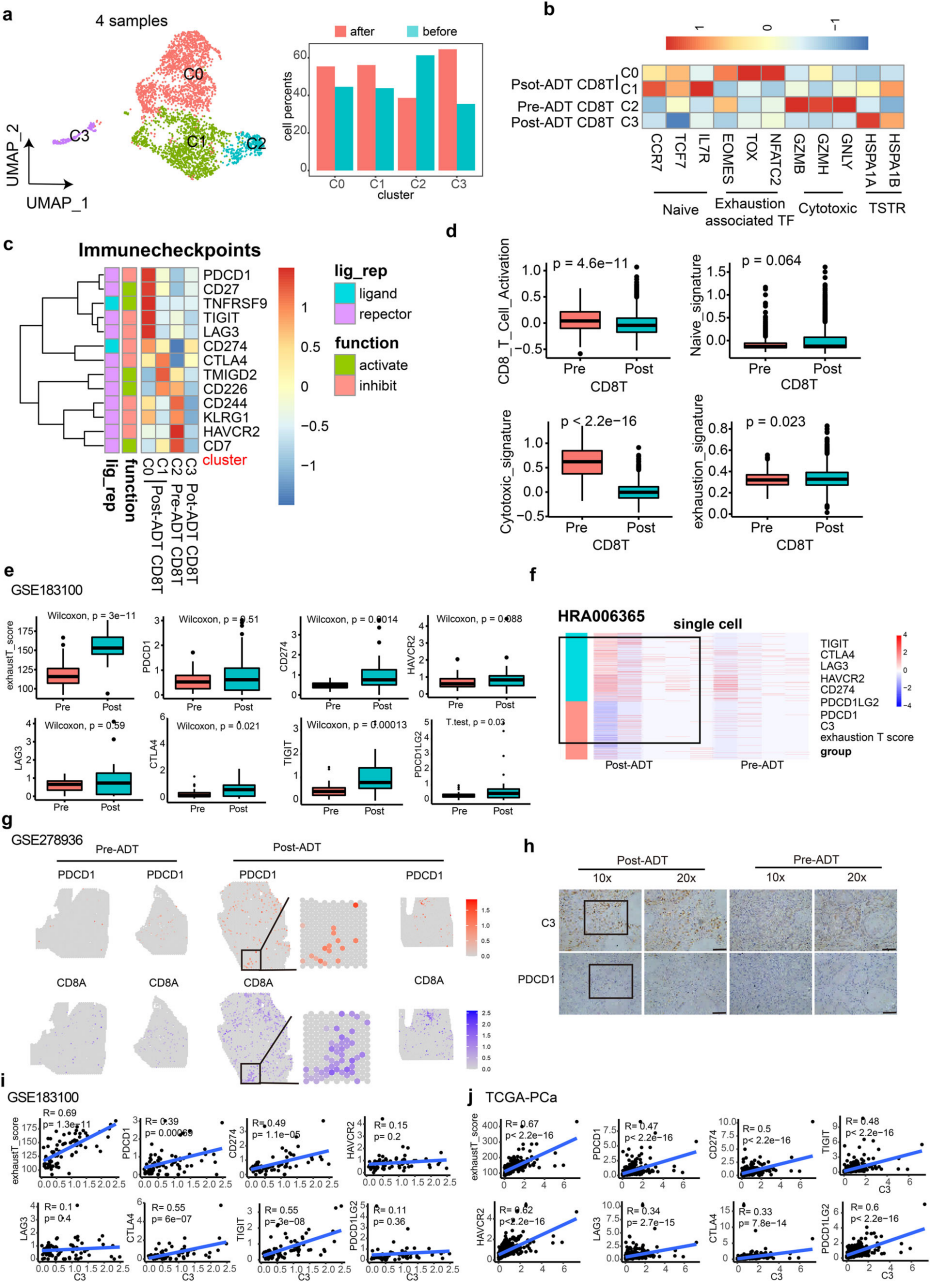
ADT promotes CD8^+^ T cell exhaustion and immune checkpoint activation. (a) Re‐clustering of CD8^+^ T cells from two PCa specimens before and after ADT identified distinct T cell subpopulations and reveals treatment‐associated compositional shifts. (b) Differential gene expression analysis of CD8^+^ T cells across treatment conditions highlighted altered activation‐ and effector‐associated programs and identifies the emergence of a stress‐responsive T cell subset (T_STR) following ADT. (c) Expression profiles of immune checkpoint molecules in CD8^+^ T cells demonstrated increased inhibitory receptor expression after ADT, suggesting enhanced T cell exhaustion. (d) Functional state scoring of CD8^+^ T cells, including naïve T cell signature, cytotoxic T cell signature, exhaustion signature, and CD8 T cell activation score. Statistical comparisons of exhaustion signatures were performed using a two‐tailed Student's *t*‐test, whereas all other comparisons were conducted using the Wilcoxon rank‐sum test. (e) Bulk RNA‐seq analysis showing increased exhaustion scores and upregulation of immune checkpoint genes in post‐ADT tumors, corroborating single‐cell findings. (f) Independent single‐cell RNA‐seq validation confirming enrichment of exhausted CD8^+^ T cell states following ADT. (g) Spatial transcriptomic analysis demonstrating increased co‐localized expression of CD8A and PDCD1 after ADT, supporting spatial expansion of exhausted T cells within the tumor microenvironment. (h) Immunohistochemical analysis revealing increased accumulation of exhausted T cells in proximity to C3‐high regions, suggesting a spatial association between complement activation and T cell dysfunction. (i, j) Correlation analyses showing positive associations between C3 expression and exhausted T cell scores, as well as immune checkpoint gene expression, indicating that C3‐driven microenvironmental remodeling may contribute to ADT‐induced T cell exhaustion.

Following ADT, however, CD8^+^ T cells underwent a profound phenotypic shift. There was marked upregulation of inhibitory immune checkpoints, including PDCD1, LAG3, CTLA4, and TIGIT (Figure 6c), accompanied by a substantial decline in cytotoxic and activation‐associated genes and a concomitant increase in exhaustion‐associated signatures (Figure 6d). These changes collectively reflect a transition toward a functionally compromised and exhausted state. Both bulk and single‐cell RNA sequencing analyses independently confirmed the accumulation of exhaustion‐related signaling and the sharp induction of inhibitory checkpoint molecules (Figure 6e,f). Spatial transcriptomic data further demonstrated increased expression and spatial enrichment of PDCD1 and CD8A following ADT (Figure 6g), indicating expansion of exhausted CD8^+^ T cells within the tumor niche. Consistently, immunohistochemical analyses revealed an increased number of exhausted T cells localized in proximity to C3‐high regions (Figure 6h). The positive correlation between C3 abundance and inhibitory checkpoint expression (Figure 6i,j) suggests that this stromal‐associated axis may actively promote T cell dysfunction. Notably, these alterations paralleled the macrophage reprogramming described above, together supporting a coordinated, therapy‐induced remodeling of the tumor immune microenvironment.

Together, these findings demonstrate that ADT not only attenuates CD8^+^ T cell cytotoxic function but also actively drives their exhaustion, thereby reinforcing systemic immune dysfunction. This concurrent remodeling of macrophages and T cells identifies immune suppression as a central limitation of ADT and provides a compelling mechanistic rationale for combining ADT with immune checkpoint blockade strategies.

### 2.6 Potential Pre‐Existing Malignant Epithelial Cells With Intrinsic Hormonal Resistance

Single‐cell transcriptomic profiling revealed a strikingly divergent response of stromal and epithelial compartments to ADT. Whereas fibroblasts expanded and acquired immunoregulatory phenotypes, epithelial populations (EPs) underwent a pronounced contraction, with most malignant clusters effectively eliminated. Despite this overall depletion, a rare yet transcriptionally distinct epithelial subset persisted, raising the possibility that a pre‐existing intrinsically resistant clone existed prior to treatment rather than emerging de novo under therapeutic pressure.

To explore this hypothesis, we compared the transcriptional landscapes of pre‐treatment tumors. Most EP clusters from PCa1 and PCa2 exhibited broadly comparable gene expression programs, consistent with shared malignant architectures across patients. In contrast, a discrete epithelial population—Cluster 16 in PCa2—displayed molecular features that correlated exclusively with Cluster 14 in PCa1 (Figure 2d). This cross‐patient concordance defined a transcriptionally coherent yet distinct malignant lineage, suggesting that intrinsic resistance may reflect a conserved epithelial state rather than merely inter‐patient heterogeneity.

Genomic profiling further substantiated this interpretation. Copy number variation (CNV) analysis demonstrated that PCa2 harbored a substantially higher genomic burden than PCa1 prior to therapy (Figure 7a). Although both tumors displayed canonical alterations on chromosomes 8 and 16, hallmarks of recurrent PCa‐associated events [21], PCa2 uniquely exhibited additional aberrations involving chromosomes 8, 9, and 13, genomic changes previously associated with aggressive disease behavior [22, 23]. These findings indicate that PCa2 was enriched for a genomically unstable, high‐risk epithelial population, thereby providing a plausible substrate for therapy resistance.

**FIGURE 7:**
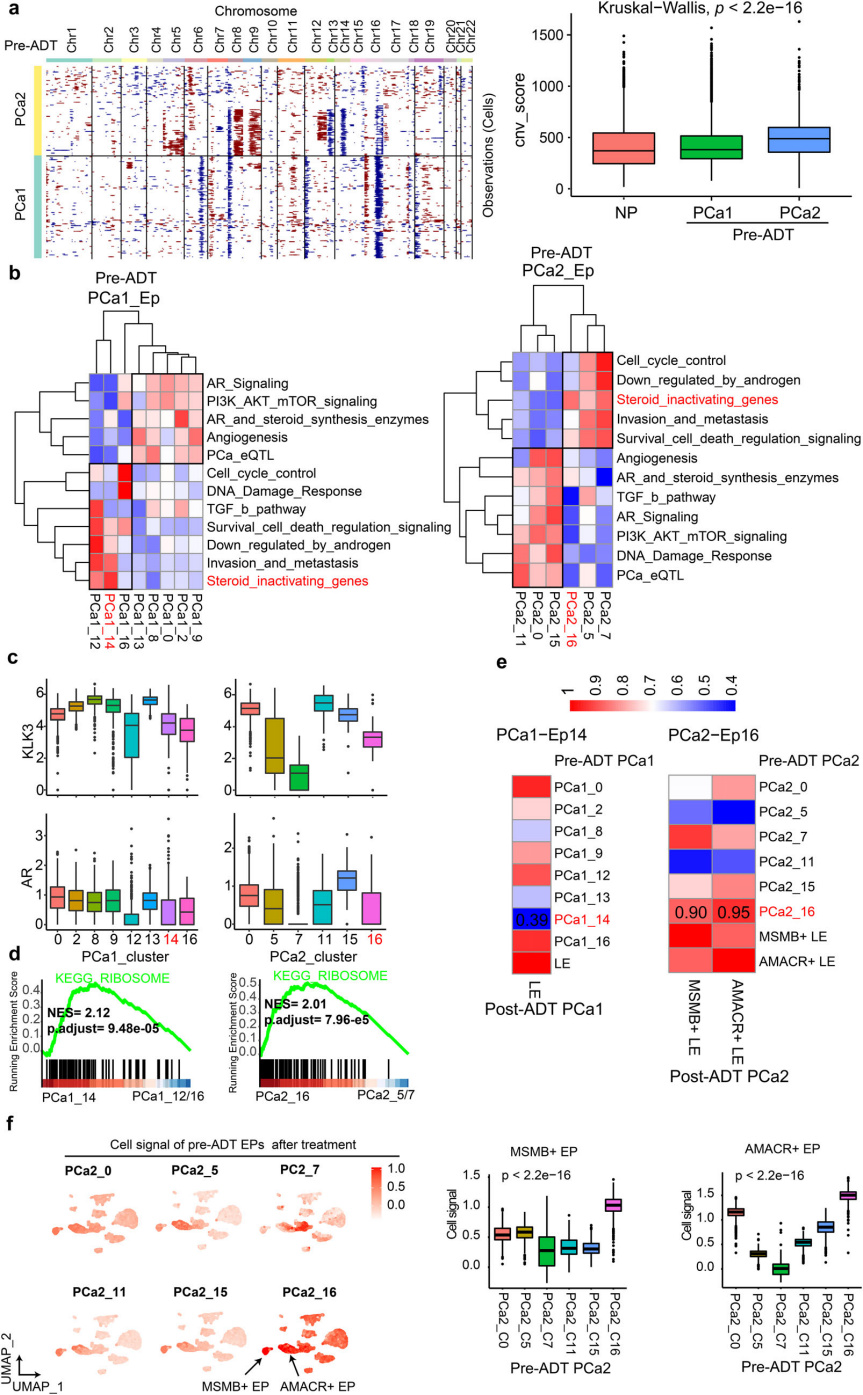
Identification of pre‐existing malignant luminal epithelial population that persisted after ADT. (a) Copy number variation (CNV) inference analysis of epithelial cells from two PCa samples prior to treatment reveals recurrent genomic alterations consistent with malignant transformation. (b) Gene Set Variation Analysis (GSVA) of prostate cancer‐related pathways in pre‐ADT samples demonstrates activation of oncogenic and proliferation‐associated signaling programs across epithelial subpopulations. (c) Expression profiling of AR and KLK3 across epithelial clusters before ADT highlights heterogeneity in androgen signaling activity and identifies AR‐low epithelial populations with potential malignant features. (d) GSEA reveals consistently activated ribosome biogenesis and translational signaling in cluster 14 (PCa1) and cluster 16 (PCa2) compared with other AR‐low epithelial populations, suggesting enhanced biosynthetic capacity in these subsets. (e) Correlation analysis (r^2^) between epithelial populations before and after ADT demonstrates preservation of specific malignant clusters following treatment. Heatmap intensity (red) corresponds to higher correlation coefficients, indicating greater transcriptional similarity and lineage persistence. (f) Projection of pre‐ADT epithelial signaling signatures onto post‐ADT samples shows sustained enrichment of cluster 16–associated programs, supporting the persistence of a pre‐existing malignant epithelial population after ADT.

To determine whether these genomic distinctions translated into functional divergence, we performed gene set variation analysis (GSVA). The transcriptionally distinct EP subsets preferentially activated pathways associated with steroid inactivation, invasion, metastasis, and cell survival (Figure 7b). Importantly, Cluster 14 EPs in PCa1 and Cluster 16 EPs in PCa2 exhibited significantly reduced expression of KLK3 and AR relative to other epithelial populations (Figure 7c), indicating diminished androgen dependency. Concurrent enrichment of ribosome‐related signaling pathways (Figure 7d) suggested enhanced translational capacity, potentially enabling sustained protein synthesis and survival under androgen‐deprived conditions. Collectively, these transcriptional and genomic features converge on an intrinsically hormone‐insensitive phenotype poised for persistence under ADT.

Consistent with this model, only a limited number of epithelial clusters persisted following treatment—one in PCa1 and two in PCa2 (Figure 3b). Strikingly, Cluster 16 EPs in PCa2 showed the strongest transcriptional correlation with the residual post‐ADT epithelial population (Pearson r^2^ = 0.90 and 0.95) (Figure 7e). Furthermore, the pre‐ADT Cluster 16 gene signature was most highly enriched within post‐treatment residual (Figure 7f), implicating this population as the direct precursor of therapy‐resistant clones. Clinically, PCa2 maintained persistently elevated serum tPSA levels after treatment, providing functional evidence that these resistant epithelial cells contributed to ongoing tumor activity. Collectively, these findings support the hypothesis that a pre‐existing subset of malignant EPs may possess intrinsic resistance to androgen suppression and may serve as a cellular reservoir that seeds relapse and contributes to CRPC progression.

### 2.7 TSPAN1 Contributes to CRPC Progression Following ADT

To delineate the malignant potential of epithelial populations that persist after ADT, we first characterized the genomic landscape of AMACR^+^ EPs in PCa2. These cells exhibited extensive chromosomal alterations involving chromosomes 8, 9, 13, and 16 (Figure 8a), indicative of pronounced genomic instability. Compared with other EP subtypes, AMACR^+^ cells displayed a markedly elevated CNV burden, further supporting their malignant identity. This genomic instability was accompanied by high KLK3 expression (Figure 8b), a well‐established marker of PCa progression. Importantly, analysis of independent patient cohorts demonstrated that AMACR^+^ EP marker genes were significantly upregulated in both primary tumors and metastatic lesions compared to normal prostate tissue (Figure 8c; Figure S7a). Together, these findings position AMACR^+^ EPs as a clinically relevant reservoir of malignant epithelial cells with the capacity to sustain disease progression under androgen deprivation.

**FIGURE 8:**
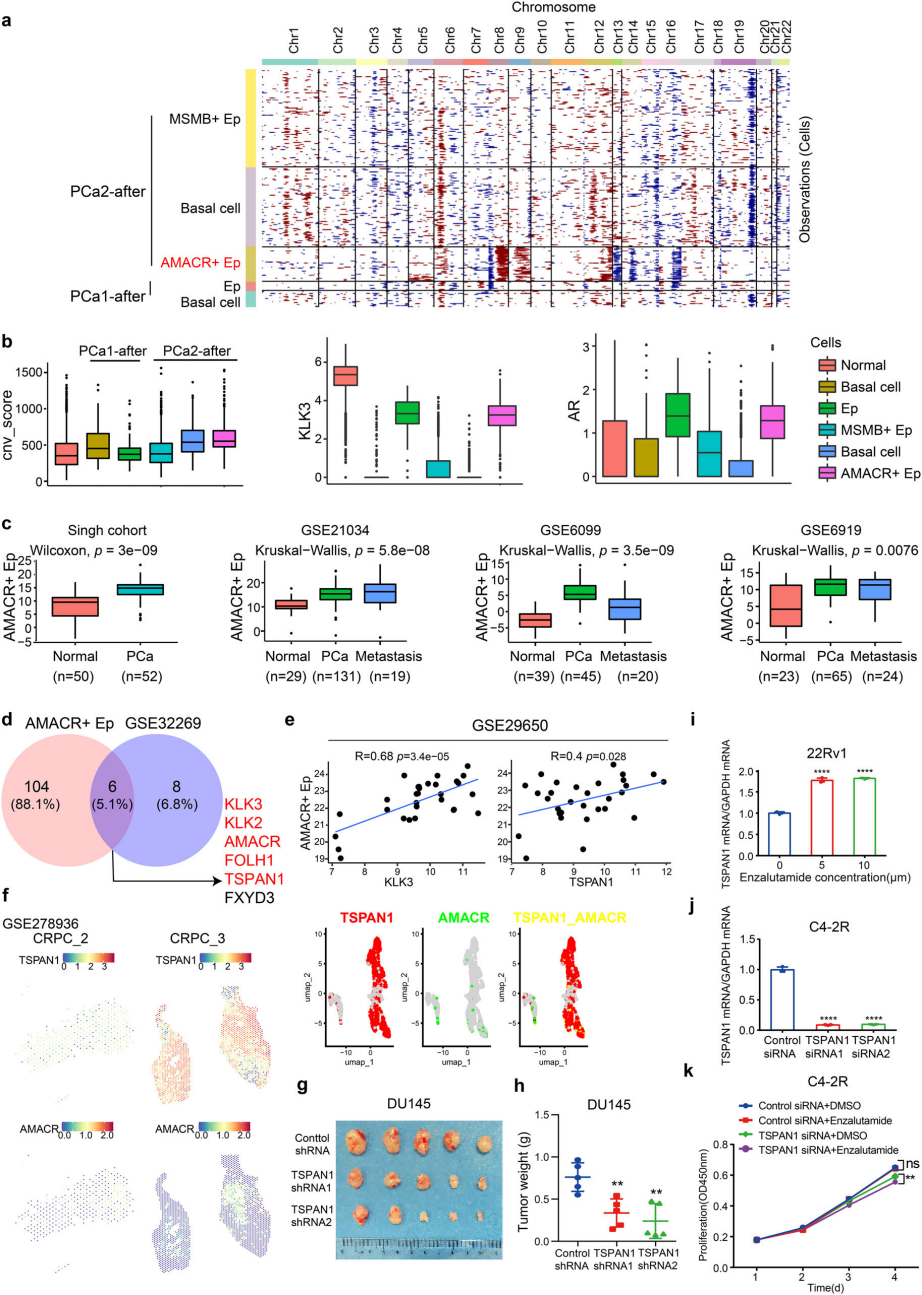
*TSPAN1* and its association with castrate‐resistant prostate cancer development. (a) Copy number variation (CNV) profiling of epithelial populations (EPs) following ADT, revealing genomic alterations associated with therapeutic resistance. (b) Integrated analysis of CNV scores and the expression levels of AR and KLK3 across epithelial population, illustrating the relationship between genomic instability and androgen receptor signaling activity. (c) Distribution and relative abundance of AMACR^+^ epithelial populations in normal prostate tissue, primary PCa, and metastatic lesions, demonstrating their progressive enrichment during disease advancement. (d) Intersection analysis of differentially expressed genes (DEGs) identified in AMACR^+^ EPs with genes associated with CRPC, highlighting a shared transcriptional program potentially driving resistance. (e) Correlation analysis between AMACR^+^ EP abundance and the CRPC‐associated genes identified in panel d, revealing key molecular candidates linked to resistance progression. (f) Spatial transcriptomic analysis of two CRPC samples identified the presence of TSPAN1+/AMACR+ double‐positive cells. (g, h). In vivo validation of TSPAN1 function. Downregulation of TSPAN1 markedly impairs tumor growth and tumorigenic capacity in xenograft models. (i–k). TSPAN1 was further validated as a critical contributor to CRPC progression. Enzalutamide treatment increased TSPAN1 gene expression (j); whereas TSPAN1 knockdown restored sensitivity to enzalutamide in resistant cells.

Having established the malignant characteristics of AMACR^+^ EPs, we next sought to identify oncogenic drivers associated with CRPC emergence. Interrogation of the GSE32269 cohort revealed six genes consistently enriched in CRPC: KLK3, KLK2, AMACR, FOLH1, TSPAN1, and FXYD3 (Figure 8d). While KLK3, KLK2, and FOLH1 are established AR‐responsive PCa biomarkers, integrative analysis of a CRPC‐specific dataset revealed that KLK3 and TSPAN1 displayed the strongest positive correlations with the AMACR^+^ epithelial transcriptional program (Figure 8e). Consistently, TSPAN1^+^/AMACR^+^ double‐positive cells were detected in the spatial transcriptomic profiles of two CRPC samples (Figure 8f). These findings suggest that, beyond canonical AR‐driven markers, a distinct epithelial substate marked by TSPAN1 expression may be functionally coupled to malignant progression under androgen‐deprived conditions.

Notably, bulk transcriptomic analyses indicated that TSPAN1 expression decreased in PCa tissues following ADT (Figure S7b), seemingly contradicting its enrichment in CRPC‐associated epithelial states. However, single‐cell resolution reconciled this discrepancy. TSPAN1 expression was preferentially elevated in mature epithelial populations but reduced in stromal compartments and proliferative epithelial subsets, including basal and Ki67^+^ cells (Figure S7c). Thus, opposing compartment‐specific dynamics likely obscured its epithelial‐specific upregulation in bulk analyses, contributing to prior interpretations of TSPAN1 as a putative tumor suppressor lacking prognostic relevance. In contrast, isolated PCa cell lines (VCaP, C4‐2B, and LNCaP) demonstrated robust TSPAN1 upregulation during CRPC induction (Figure S7d). Elevated TSPAN1 expression was further observed in subsets of primary and metastatic tumors (Figure S7e), predominantly within adenocarcinoma rather than neuroendocrine PCa contexts (Figure S7f), and was significantly associated with biochemical recurrence (Figure S7g). Together, these findings redefine TSPAN1 as a lineage‐restricted, context‐dependent oncogenic factor enriched within the AMACR^+^ epithelial compartment during CRPC progression.

Functional validation substantiated this model. Stable TSPAN1 knockdown in multiple PCa cell lines (22Rv1, DU145, PC3, and LNCaP) significantly impaired cell proliferation, clonogenic growth, and migratory capacity in vitro (Figure S7h–w). Consistently, DU145 xenografts with TSPAN1 silencing formed substantially smaller tumors in vivo, as evidenced by reduced tumor volume and weight compared with controls (Figure 8g,h). In addition, enzalutamide treatment significantly increased TSPAN1 expression (Figure 8i). Notably, knockdown of TSPAN1 in the enzalutamide‐resistant C4‐2R cell line restored sensitivity to enzalutamide, thereby enhancing its tumor‐inhibitory effect (Figure 8j,k). These results establish TSPAN1 as a functional driver of malignant growth in PCa. Importantly, gene regulatory network (GRN) analysis revealed that the upstream regulatory circuitry controlling TSPAN1 closely resembled AR‐positive networks characterized by active FOXA1 (Figure S8) [24], situating TSPAN1 within an AR‐FOXA1‐driven epithelial regulatory framework.

Taken together, our findings define TSPAN1 as a critical effector of AMACR^+^ epithelial oncogenic potential and a previously underappreciated contributor to CRPC progression, highlighting its potential as a lineage‐informed therapeutic target.

### 2.8 NRXN1 Associates With Prostate Cancer Progression and Mediates CRPC‐NE Emergence Through Calcium Signaling Rewiring

In parallel with the persistence of adenocarcinoma‐associated programs, single‐cell transcriptomic profiling uncovered an emergent epithelial cluster enriched for neuroendocrine signatures in patient tumors following ADT. This population was characterized by suppression of AR signaling, activation of epithelial‐mesenchymal transition (EMT) pathways, and enrichment of canonical neuroendocrine transcriptional programs (Figures 3b–d and 9a; Figures S8 and S9) [23], features consistent with the molecular hallmarks of NEPC. These data indicate that therapeutic pressure promotes a lineage shift toward a more plastic, aggressive, and treatment‐refractory cellular state. Importantly, GRN analysis demonstrated that this transition was not driven by the simple activation of a pre‐existing, static NE module; rather, it reflected progressive ADT‐induced regulatory rewiring, suggesting the engagement of dynamic upstream mediators.

**FIGURE 9:**
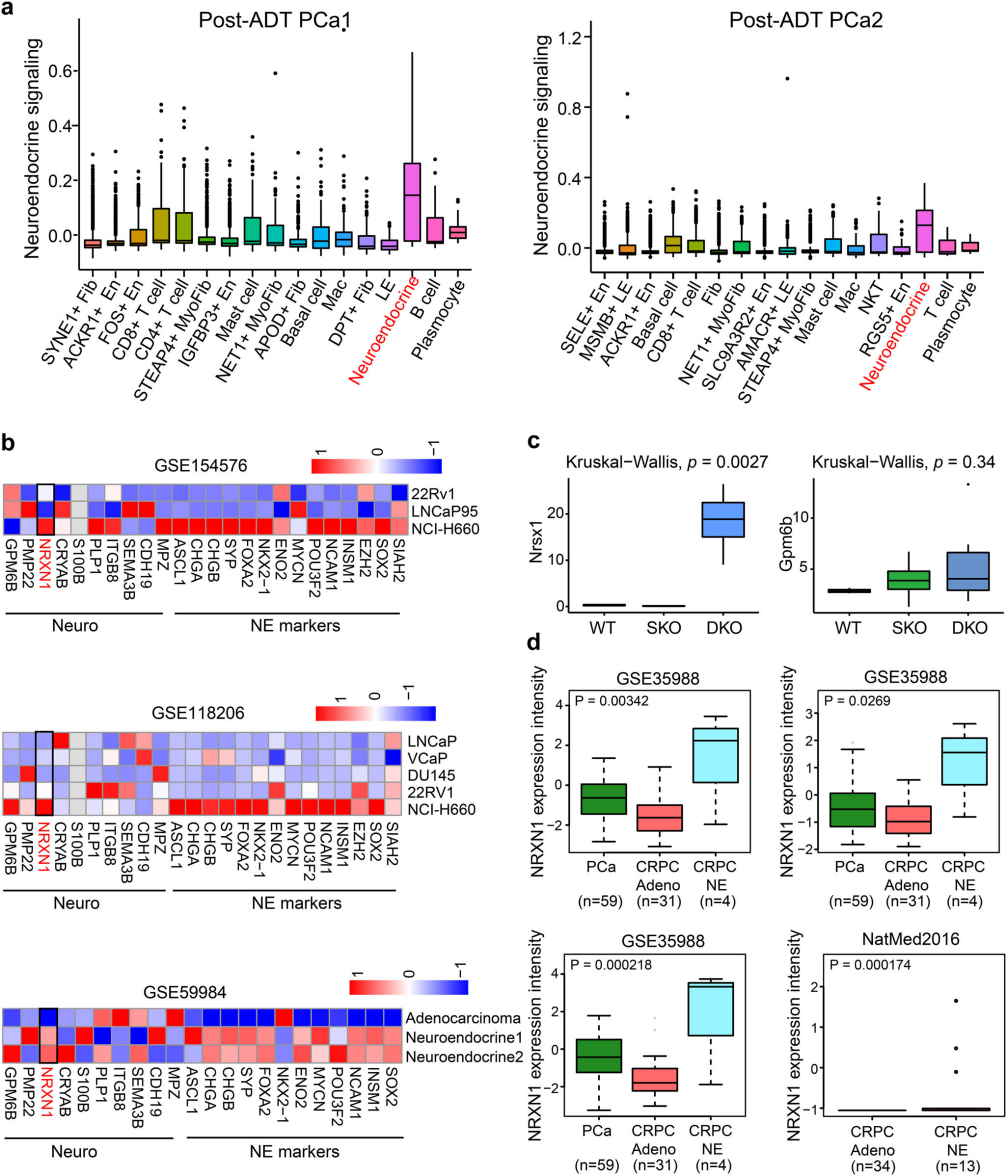
*NRXN1* expression and its association with neuroendocrine‐type castration‐resistant prostate cancer (CRPC‐NE). (a) Neuroendocrine signaling scores across individual cell clusters following ADT, revealing the emergence and enrichment of neuroendocrine‐like features within specific epithelial subpopulations under therapeutic pressure. (b) Identification of differentially expressed genes (DEGs) and established NEPC markers in NEPC‐representative cell lines and tumor xenograft models, defining transcriptional programs associated with neuroendocrine lineage plasticity and disease progression. (c) Gene expression profiling in transgenic mouse models harboring single knockout (SKO) or double knockout (DKO) of RB1 and TP53, demonstrating that combined loss of these tumor suppressors enhanced NRXN1 expression and promoted neuroendocrine differentiation. (d) Comparative analysis of NRXN1 expression in prostate adenocarcinoma and NEPC, highlighting its upregulation in NEPC and supporting its potential utility as a marker of lineage transition and aggressive disease behavior.

To systematically identify candidate mediators of this lineage transition, we interrogated three independent bulk RNA‐seq datasets (GSE154576, GSE118206, GSE59984) comprising PCa cell lines and xenograft models. Comparative analyses between NEPC and non‐neuroendocrine models confirmed robust upregulation of canonical NE markers (SYP, CHGA, CHGB) in NEPC contexts. Strikingly, NRXN1 consistently mirrored this NE‐associated expression pattern across all datasets (Figure 9b). Furthermore, in a genetically engineered mouse model harboring dual TP53 and RB1 loss—genetic alterations known to promote neuroendocrine plasticity—NRXN1 expression was significantly induced (p = 0.0027; Figure 9c). Together, these convergent transcriptional and genetic data establish a strong association between NRXN1 activation and CRPC‐NE development, positioning NRXN1 as a putative mediator of therapy‐induced lineage plasticity.

Clinical analyses further reinforced this association. NRXN1 expression was significantly elevated in primary and metastatic PCa compared with benign prostate tissues, with the highest levels observed in NEPC specimens (Figure S10a–d; Figure 9d). Elevated NRXN1 was enriched in high‐risk disease subgroups (Figure S10e–g) and independently predicted shorter biochemical recurrence‐free survival (HR = 1.965, p = 0.048; Figure S10h). Longitudinal transcriptomic datasets demonstrated increased NRXN1 expression following ADT (Figure S10i), progressive upregulation during transition to CRPC (Figure S10j), and sustained elevation in metastatic lesions (Figure S10k). Notably, NRXN1 levels were significantly higher in NEPC compared with adenocarcinoma samples (Figure S10l), supporting a role in ADT‐driven neuroendocrine transformation during disease evolution.

To gain mechanistic insight, correlation analyses were performed across bulk and single‐cell datasets. NRXN1 expression positively correlated with established neuroendocrine markers (CHGA, CHGB, ENO2, SOX2) as well as pro‐angiogenic factors (VEGFA, FGF2, PDGFB), suggesting involvement in both lineage plasticity and microenvironmental remodeling (Figure S11). In contrast, NRXN1 expression was uncoupled from canonical AR signaling, showing no correlation with AR or KLK3. Instead, NRXN1 exhibited associations with both pro‐ and anti‐apoptotic regulators (BAX, BCL2), implying broader roles in cellular survival regulation (Figure S11). Integrative scoring analyses further demonstrated a negative correlation between NRXN1 expression and AR activity, alongside a positive association with NEPC signature scores (Figure S12). Collectively, these data position NRXN1 as both a functional marker and a potential regulatory node in the CRPC‐NE transition.

We next investigated whether NRXN1 plays a causal role in lineage plasticity. Hormonal manipulation experiments revealed that androgen deprivation induced NRXN1 expression in LNCaP cells, whereas androgen supplementation suppressed NRXN1 in 22Rv1 and VCaP cells (Figures S10j and S13a,b), indicating hormonal responsiveness and inverse coupling with AR activity. Functional perturbation studies further demonstrated that NRXN1 silencing in NE1.3, PC3, and LNCaP cells significantly impaired proliferation, clonogenic growth, migration, and in vivo tumor expansion (Figure 10a–f; Figure S13c–h). Moreover, NRXN1 depletion reduced sphere formation in 3D cultures (Figure 10g), implicating NRXN1 in stemness‐associated phenotypes.

**FIGURE 10:**
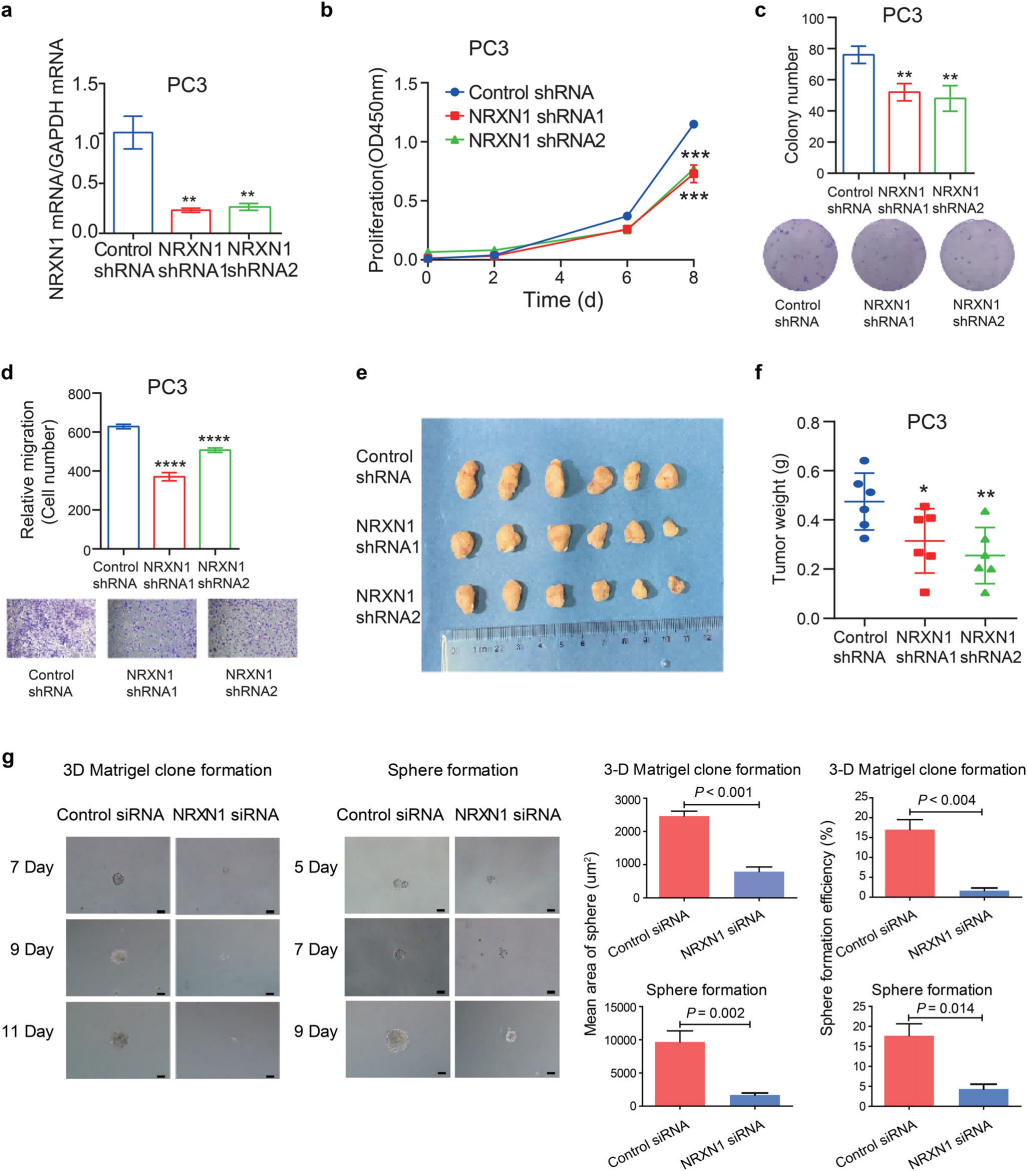
*NRXN1* promotes prostate cancer progression and tumorigenic potential. (a–d) In vitro functional assays demonstrating that (a) NRXN1 knockdown significantly suppresses (b) cell proliferation, (c) clonogenic growth, and (d) migratory capacity, indicating that NRXN1 contributes to aggressive tumor phenotypes. (e, f) In vivo validation in xenograft models showing that downregulation of NRXN1 significantly impairs tumor growth and tumorigenic potential, confirming its functional importance in CRPC‐NE development and disease progression. (g) Three‐dimensional growth assays further reveal that silencing *NRXN1* markedly reduces sphere formation and 3D Matrigel colony formation in C4‐2 cell lines, supporting its role in maintaining tumor cell stemness and growth capacity under physiologically relevant conditions.

Prolonged androgen deprivation generated CRPC‐NE LNCaP cells exhibiting elevated NRXN1 expression, neurite‐like projections, and induction of NEPC markers (SYP, CHGB, ENO2) (Figure 11a). Notably, NRXN1 knockdown attenuated synapse‐like structural formation and reduced NE gene expression, as confirmed by immunofluorescence analyses (Figure 11b,c). Consistently, patient‐derived NEPC tissues displayed strong NRXN1 staining compared with adenocarcinoma samples (Figure S13i). Moreover, activation of NRXN1 may be associated with increased expression of its ligand NLGN1 (Figure S13j). These findings support a functional role for NRXN1 in promoting neuroendocrine transdifferentiation under ADT pressure.

**FIGURE 11:**
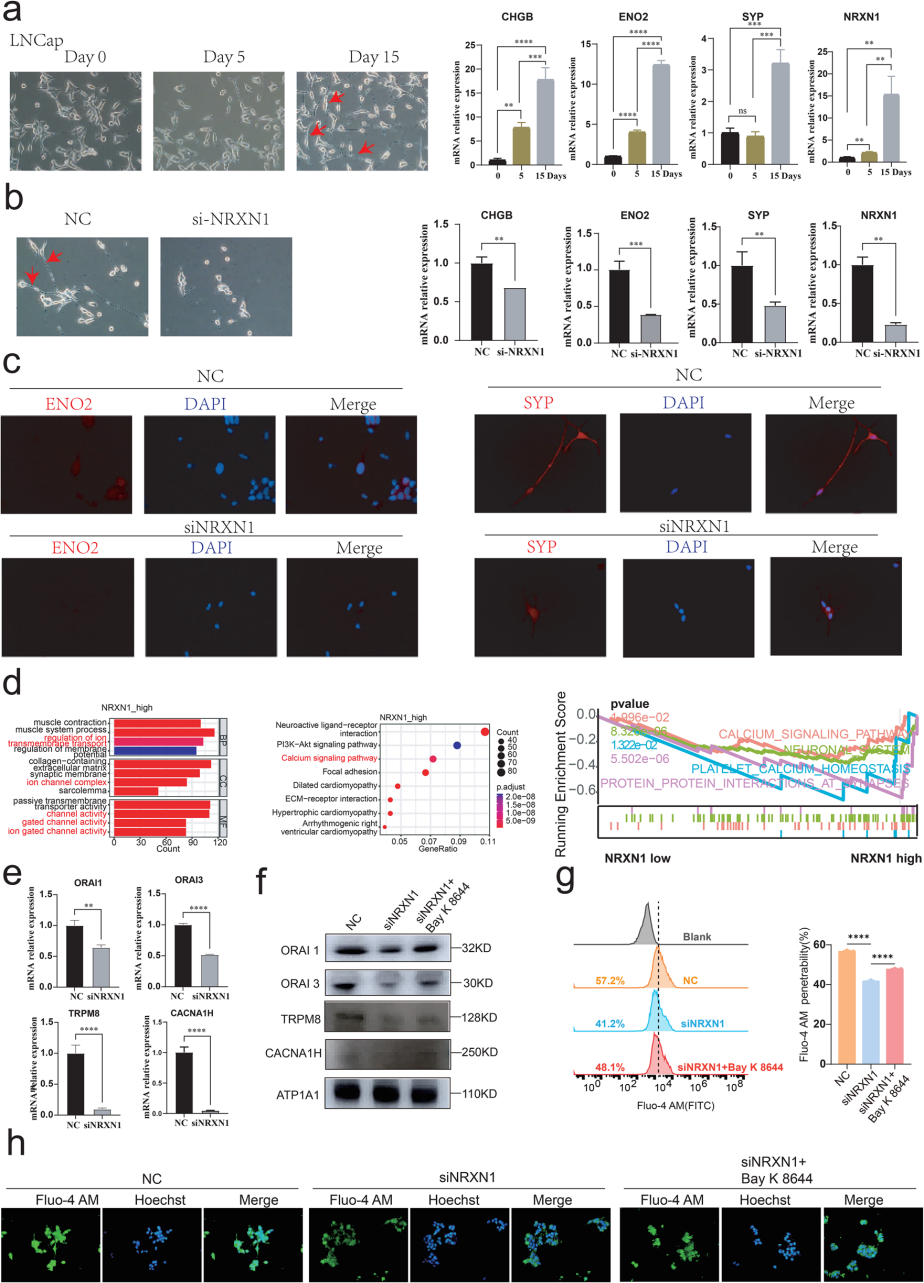
*NRXN1* induces neuroendocrine phenotypes through calcium channel activation during CRPC‐NE progression. (a) Establishment of CRPC‐NE cellular models exhibiting elevated expression of neuroendocrine markers and NRXN1, providing an experimental platform to investigate lineage plasticity mechanisms. (b, c) Suppression of NRXN1 reversed neuroendocrine features, as evidenced by reduced expression of neuroendocrine markers and attenuation of NE‐like phenotypes, indicating that NRXN1 is required for maintenance of the neuroendocrine state. (d–f) Mechanistic analyses demonstrating that NRXN1 regulated calcium channel activation during CRPC‐NE progression, linking NRXN1‐driven signaling to intracellular calcium dynamics and neuroendocrine differentiation programs. (g, h) Suppression of NRXN1 inhibited the intracellular calcium ion influx, demonstrating the impaired function of calcium channels.

To delineate downstream mechanisms, pathway enrichment analyses were performed in NRXN1‐high vs. NRXN1‐low contexts. GO, KEGG, and GSEA consistently identified calcium signaling as a significantly enriched pathway (Figure 11d). Key components of calcium flux machinery, including stromal interaction molecules (STIM), ORAI family members, transient receptor potential (TRP) channels, and voltage‐gated calcium channels (VGCCs), were markedly upregulated (Figure S13k), indicating activation of both store‐operated and voltage‐dependent calcium entry programs.

We therefore tested whether NRXN1 regulates calcium signaling at both transcriptional and functional levels. NRXN1 silencing significantly reduced expression of multiple calcium channel genes, including ORAI1, ORAI3, TRPM8, and CACNA1H (Figure 11e,f; Figure S13l), suggesting that NRXN1 coordinates calcium channel networks. Functionally, NRXN1 depletion attenuated intracellular calcium influx and altered membrane potential, as measured by flow cytometry and immunofluorescence assays (Figure 11g,h; Figure S13m,n). Importantly, pharmacologic activation of calcium channels partially rescued these defects, demonstrating that NRXN1 operates upstream of calcium channel activation and governs calcium‐dependent signaling output rather than merely modulating membrane excitability.

Collectively, our findings identify NRXN1 as a previously unrecognized upstream regulator of calcium signaling in PCa. By coupling adhesion‐associated programs to calcium influx and membrane depolarization, NRXN1 integrates microenvironmental cues with intracellular signaling dynamics. Given the established role of calcium signaling in promoting PCa progression and lineage plasticity [25], we propose a model in which ADT‐induced NRXN1 upregulation sustains calcium‐dependent signaling programs that reinforce stemness, angiogenesis, and neuroendocrine identity, thereby driving neuroendocrine transdifferentiation and therapeutic resistance.

## 3 Discussion

Therapy resistance and progression in PCa do not arise from a single molecular lesion but instead reflect coordinated remodeling across epithelial, stromal, and immune compartments [26, 27, 28]. Consistent with prior single‐cell analyses of human prostate specimens [9, 29], including cross‐sectional atlases of localized and advanced disease, we identified the canonical cellular constituents of the prostate tumor ecosystem. Notably, before ADT, epithelial cell proportions in our cohort were lower than in certain treatment‐naïve datasets [9], whereas immune infiltration, particularly T cells, was relatively enriched in tumors compared with adjacent normal tissues. These differences likely reflect variation in tumor grade, clinical stage, and inflammatory context. Importantly, however, previous studies have largely provided static snapshots of tumor composition [9]. The dynamic consequences of ADT on cell‐state transitions and ecosystem remodeling in human tumors have remained incompletely defined.

By integrating longitudinal single‐cell RNA sequencing with functional validation, we delineated how ADT reshaped the tumor microenvironment in real time. Our findings support a unifying model in which androgen deprivation induces fibroblast bifurcation, generating a DPT^+^ cancer‐associated fibroblast (CAF) population that establishes a complement‐driven macrophage axis reinforcing immune escape. Concurrently, ADT preserves malignant epithelial subpopulations that deploy TSPAN1‐ and NRXN1‐dependent adaptive programs. Together, these data reframe resistance as an ecosystem‐level process emerging from coordinated intercompartmental crosstalk rather than purely cell‐intrinsic alterations.

ADT is classically understood as a suppressor of AR signaling within tumor epithelial cells. Our analysis substantially expanded this paradigm by demonstrating profound stromal and immune reconfiguration following androgen deprivation. We uncovered marked fibroblast heterogeneity, including the emergence of APOD^+^ fibroblasts characterized by reduced AR activity and a more terminally differentiated, less inflammatory phenotype. These observations indicate that ADT does not simply attenuate stromal function but actively rewires fibroblast biology, suppressing canonical protumorigenic CAF programs while enabling alternative differentiation trajectories.

Functionally, ADT suppressed fibroblast‐derived pleiotrophin (PTN) and midkine (MDK) signaling, cytokine networks implicated in angiogenesis, stemness, and tumor progression. Attenuation of PTN/MDK activity suggests that ADT‐driven stromal remodeling may transiently restrain oncogenic signaling, thereby contributing to the initial therapeutic response [30, 31, 32]. These data support a temporally dynamic model in which ADT exerts dual effects on the TME: early suppression of protumorigenic stromal circuits followed by progressive activation of adaptive microenvironmental programs that facilitate immune evasion and disease persistence.

Consistent with its central role in PCa management, ADT has been reported to enhance immunosuppressive ecological features within the TME through broad stromal and immune remodeling [33]. Extending this concept, we identified a previously unrecognized expansion of DPT^+^ fibroblasts with pronounced immunoregulatory properties following ADT. These cells exhibited elevated C3 expression, implicating complement activation as a key mediator of stromal‐driven immune modulation. Mechanistically, C3 signaling promotes macrophages polarization toward an M2‐like phenotype while exacerbating T cell exhaustion. Thus, DPT^+^ fibroblasts emerge as critical orchestrators of ADT‐associated immunosuppression.

Importantly, this ADT‐induced fibroblast population is mechanistically distinct from the previously described immunosuppressive FerroCAFs in PCa [17]. FerroCAFs appear constitutively present in untreated tumors and are characterized by disrupted iron homeostasis and ferroptosis‐associated pathways [18]. Therapeutic targeting of the HMOX1/iron/KDM6B axis restores intracellular iron balance in FerroCAFs and reinvigorates antitumor immunity [17], highlighting the immunological consequences of iron dysregulation in the stromal compartment. In contrast, the DPT^+^ CAF subset uncovered here operates through a complement‐driven immunomodulatory axis rather than an iron‐dependent ferroptotic program. This distinction underscores that stromal immunoregulation in PCa is temporally layered and mechanistically heterogeneous: baseline iron‐dependent suppression coexists with therapy‐induced complement activation under androgen deprivation. Together, these findings expand the conceptual framework of stromal plasticity and position fibroblast subsets as rational targets for combinatorial strategies aimed at disrupting multiple immunosuppressive axes.

Beyond stromal reprogramming, ADT profoundly reshaped the immune landscape itself. Androgen deprivation alters the composition of tumor‐infiltrating immune populations and promotes T cell dysfunction, as evidenced by elevated expression of exhaustion markers including PDCD1, LAG3, and CTLA4. These observations provide mechanistic support for clinical strategies combining AR antagonism with immune checkpoint blockade to restore T cell activity and prolong therapeutic benefit [34, 35, 36]. Importantly, our single‐cell‐resolved analyses directly capture the transition toward immune exhaustion in patient‐derived tumors, strengthening the biological rationale for combinatorial immunohormonal interventions.

Despite extensive remodeling of stromal and immune compartments, a subset of malignant epithelial cells persisted following androgen deprivation. In particular, AMACR^+^ epithelial populations enriched for copy number alterations are retained after treatment. These clones activate steroid‐inactivating pathways while maintaining KLK3 expression, suggesting adaptive fitness under androgen‐depleted conditions and a potential role in driving progression toward CRPC [11, 12, 37, 38]. The persistence of these therapy‐tolerant epithelial lineages highlights a fundamental limitation of androgen suppression alone and underscores the necessity of strategies capable of eradicating resistant subclonal populations.

Building upon this framework of epithelial persistence and adaptive survival, our integrative multi‐omics analyses identified TSPAN1 and NRXN1 as candidate drivers of therapeutic resistance in advanced PCa. Although TSPAN1 has been variably reported as oncogenic or tumor‐suppressive in PCa [39, 40, 41], accumulating evidence across malignancies supports a predominantly protumorigenic role. Functional studies implicate TSPAN1 in regulating cell proliferation, epithelial‐mesenchymal transition, stemness maintenance, angiogenesis, autophagy, chemoresistance, and microenvironmental remodeling [42]. Our longitudinal analyses further demonstrate progressive upregulation of TSPAN1 during CRPC evolution, accompanied by associations with hormone insensitivity and enhanced migratory capacity. Rather than representing a PCa‐specific anomaly, these findings position TSPAN1 as a context‐responsive mediator of adaptive resistance. Its established role in organizing membrane microdomains and modulating signal transduction provides a plausible framework through which it may influence AR signaling dynamics and downstream resistance programs.

In parallel, NRXN1 emerges as an ADT‐induced factor associated with neuroendocrine differentiation and adverse clinical outcomes, implicating it as a previously underappreciated regulator of tumor plasticity. Together, these data identify TSPAN1 and NRXN1 as mechanistically grounded biomarkers of resistance and candidate therapeutic targets in advanced PCa.

A major strength of this study lies in its longitudinal design. Prior investigations utilizing patient‐derived xenograft models [43, 44] or cross‐sectional patient cohorts [9, 45] have provided valuable insights into resistance biology but were inherently limited in resolving dynamic, therapy‐induced state transitions within human tumors. By constructing a longitudinal single‐cell atlas of patient‐derived PCa under ADT, we establish a framework to directly chart ecosystem‐level remodeling in a clinically relevant context. This systems‐level perspective enables reconstruction of resistance trajectories and informs rational therapeutic design grounded in temporal tumor evolution.

Collectively, our findings advocate for treatment paradigms that extend beyond epithelial‐centric targeting to simultaneously: (i) eliminate resistant malignant clones, (ii) disrupt fibroblast‐immune crosstalk, and (iii) integrate immunomodulatory strategies with hormonal blockade to counteract adaptive resistance mechanisms operating across compartments.

In summary, we demonstrate that ADT initiates a fibroblast‐derived complement program that suppresses antitumor immunity while coordinating epithelial resistance through distinct molecular effectors. By reframing therapy resistance as an ecosystem‐level process rather than a purely cell‐intrinsic phenomenon, our study identifies the complement axis, TSPAN1, and NRXN1 as translationally actionable vulnerabilities. More broadly, these findings underscore that PCa progression reflects coordinated interactions among stromal, immune, and epithelial compartments, thereby providing both a conceptual framework and therapeutic foundation for ecosystem‐targeted intervention strategies.

## 4 Materials and Methods

### 4.1 Patient Cohort

This study included prostate tissue specimens from five individuals with histologically normal prostates, four patients with prostate cancer (PCa), and two patients with PCa who underwent follow‐up biopsies during the course of ADT. Among the four PCa cases, two patients presented to our institution with markedly elevated serum total prostate‐specific antigen (tPSA) levels (>1000 ng/mL) at initial diagnosis. Both individuals were treatment‐naïve, and subsequent comprehensive clinical evaluations—including imaging studies and laboratory analyses—demonstrated metastatic disease with confirmed bone and pelvic lymph node involvement.

To establish the diagnosis, each patient underwent a 12–14 core transperineal systematic prostate biopsy, with histopathological examination confirming the presence of adenocarcinoma (Table S1). For longitudinal analysis, repeat biopsies were performed under ultrasound guidance, targeting the same anatomical regions as the initial sampling to ensure consistency and comparability.

For single‐cell RNA sequencing (scRNA‐seq), six biopsy cores were randomly selected from each patient and processed to generate high‐quality single‐cell suspensions. Written informed consent was obtained from all participants prior to enrollment. The study protocol was reviewed and approved by the Medical Ethics Committee of Guangxi Medical University (Approval Number: 2022‐KY‐(054)), and all procedures adhered to the principles of the Declaration of Helsinki.

### 4.2 Single‐Cell Suspension

Fresh prostate biopsy tissues were immediately immersed in chilled transfer buffer (RPMI medium supplemented with 1% antibiotic/antimycotic solution) and delivered to the laboratory within 15 min of collection. Approximately 1 g of tissue per sample was transferred into 15‐mL conical tubes, washed with Dulbecco's Phosphate‐Buffered Saline (DPBS), and centrifuged at 300 g for 3 min at 4°C to remove residual medium.

Tissue fragments were then subjected to enzymatic dissociation in 10 mL of digestion buffer containing collagenase type VI, dispase II, DNase I, and 10% fetal bovine serum (FBS) in Hank's Balanced Salt Solution (HBSS). Samples were incubated at 37°C for 90 min with gentle agitation to promote efficient disaggregation into single cells. Following digestion, 3 mL of 1× RBC lysis buffer (BioLegend, Cat. no. 420301) was applied to eliminate erythrocyte contamination. Cells were centrifuged, and the supernatant was carefully aspirated.

The resulting suspension was sequentially filtered through a 40‐µm cell strainer into ice‐cold DPBS to remove residual tissue debris and aggregates. The pelleted cells were washed and resuspended in DPBS, and cell viability was assessed by trypan blue exclusion using a hemocytometer. Only preparations with >85% viable cells were used for subsequent single‐cell RNA sequencing (scRNA‐seq).

### 4.3 Sequencing and Data Processing

Raw sequencing data were processed using the Cell Ranger software suite (v6.1.1; 10x Genomics). FASTQ files were generated with the cellranger mkfastq pipeline and subsequently processed with cellranger count for alignment to the human reference genome (GRCh38), barcode demultiplexing, and unique molecular identifier (UMI) quantification.

Downstream quality control and analyses were performed using the Seurat R package (v4.0.3). Cells were retained if they expressed between 200 and 6000 genes, while genes expressed in fewer than 10 cells were excluded. The percent of mitochondrial and hemoglobin gene content was lower than 25% and 10%. To reduce technical artifacts, expression matrices were also corrected for mitochondrial and hemoglobin gene content. Highly variable genes were identified using the “vst” method, and the data were scaled and normalized prior to dimensionality reduction.

Principal component analysis (PCA) was conducted, with significant components determined by the JackStraw test and ElbowPlot. The top 20 PCs were selected for downstream clustering analyses. Graph‐based clustering was performed with a resolution parameter of 0.5, optimized to capture meaningful cellular heterogeneity. Differentially expressed genes (DEGs) across clusters were identified using the FindAllMarkers function.

To ensure robust integration across samples and minimize batch effects, we applied Harmony (RunHarmony, Seurat wrapper) for dataset integration [46]. This strategy allowed re‐clustering on Harmony‐derived embeddings rather than initial PCA space, providing improved alignment of cellular populations across biological replicates and enhancing the accuracy of downstream comparative analyses.

### 4.4 Gene Module Scoring

To assess pathway activity and cell state–specific programs, gene module scores were calculated using the AddModuleScore function in Seurat. Predefined gene sets representing key epithelial lineages and functional states—including basal cell, luminal epithelium (LE), stemness, neuroendocrine differentiation, and epithelial–mesenchymal transition (EMT)—were curated from the GSE137829 dataset.

In addition, immune‐related transcriptional programs were evaluated by applying established signatures for naïve T cells, cytotoxic T cells, exhausted T cells, and CD8^+^ T‐cell activation, as described in prior studies [20, 47]. To further explore immunoregulatory pathways, we examined the expression of canonical immune checkpoint molecules (PDCD1, CD274, CTLA4, HAVCR2, PDCD1LG2, TIGIT, and LAG3) across cellular clusters. Together, these analyses enabled systematic interrogation of both epithelial lineage states and immune modulatory programs, providing insight into tumor–immune interactions within the prostate tumor microenvironment.

### 4.5 Gene Ontology (GO) Annotation

Functional enrichment analyses were conducted using the clusterProfiler R package to delineate the biological roles of transcriptionally defined cell clusters. Gene identifiers were converted to standardized human genome annotations using the org.Hs.eg.db database to ensure precise mapping. Enrichment was assessed for Gene Ontology (GO) terms in the “Biological Process” category, and the top ten significantly enriched terms for each cluster were visualized to highlight dominant cellular functions and signaling pathways. This strategy enabled systematic characterization of the molecular programs underpinning each cell population, providing insights into their potential contributions to prostate cancer progression, lineage plasticity, and therapeutic response.

### 4.6 Spatial Transcriptomics Analysis

Spatial transcriptomics data were processed and analyzed using Seurat (v5.4.0). To enable systematic comparisons across disease states, two representative samples were selected from each clinical category [45], including benign prostatic hyperplasia (BPH), treatment‐naïve primary prostate cancer (TRNA), neoadjuvant‐treated tumors (NEADT), castration‐resistant prostate cancer (CRPC), and metastatic castration‐resistant prostate cancer (MET). The selected datasets were merged for integrated downstream analyses.

Rigorous quality control criteria were applied prior to integration. Spatial spots containing between 200 and 7,500 detected features were retained, and spots with mitochondrial transcript content exceeding 20% were excluded to minimize low‐quality or stressed cell contamination. Data normalization and scaling were performed according to standard Seurat workflows.

To mitigate batch effects and improve cross‐sample comparability, Harmony was employed for dimensionality reduction and data integration. This approach enabled the identification of shared transcriptional programs across heterogeneous disease stages while preserving biologically meaningful variation.

To further resolve cellular heterogeneity within spatial spots, we applied the Robust Cell Type Decomposition (RCTD) algorithm for reference‐based cell type deconvolution. This strategy allowed us to infer the proportional composition of stromal, immune, and epithelial cell populations across spatial regions. By integrating spatial localization with cellular composition, our analysis provides a structured framework for dissecting ecosystem‐level interactions within the prostate tumor microenvironment and establishes a foundation for therapeutic strategies targeting tumor–microenvironment crosstalk.

### 4.7 Clinical Prostate Cancer Dataset Analysis

To validate and extend our findings, clinical and transcriptomic data for prostate cancer (PCa) were obtained from cBioPortal for Cancer Genomics [48], Oncomine [49], and the Gene Expression Omnibus (GEO) [50]. Samples were stratified into normal prostate, primary PCa, and metastatic PCa groups to enable comparative expression analyses. Associations between gene expression and clinical parameters were further explored according to Gleason score (≤6, 7, ≥8), tumor stage (I–IV), and serum prostate‐specific antigen (PSA) levels (4–10, 10–20, > 20 ng/mL).

For statistical analyses, the Mann–Whitney U test was used for two‐group comparisons, and the Kruskal–Wallis H test was applied for comparisons across multiple groups. Survival analyses were conducted using the *survival* R package (v2.40). Kaplan–Meier curves were generated using the survfit function, with median gene expression values employed as cutoffs. Log‐rank tests and Cox proportional hazards regression models were used to estimate hazard ratios (HRs) and assess significance.

To interrogate neuroendocrine prostate cancer (NEPC), we incorporated gene expression data from two published cohorts [51, 52]. In addition, correlation analyses between gene expression, androgen receptor (AR) signaling, and NEPC scores were performed using the dataset reported by Beltran et al. [52]. A significance threshold of *p* < 0.05 was used throughout. All computational and statistical analyses were carried out in R (v3.5.2) using RStudio (v1.1.453).

### 4.8 Gene Expression Correlation Analysis

To explore transcriptional relationships, pairwise correlations between genes of interest were assessed using Pearson's product‐moment correlation and, where appropriate, Spearman's rank correlation coefficient (ρ). These approaches were applied to evaluate the association of NRXN1 expression with other relevant genes, as well as the relationships between AR signaling activity and the NEPC score across multiple independent PCa cohorts [51, 53, 54]. The AR signaling and NEPC scoring metrics were derived from the study by Beltran et al. [52]. All correlation analyses were performed using data from previously published, clinically annotated datasets, thereby ensuring robust cross‐cohort validation and minimizing dataset‐specific bias. This framework enabled systematic interrogation of gene co‐regulation and pathway interactions within the heterogeneous molecular landscape of prostate cancer.

### 4.9 Copy Number Variation Analysis

Copy number variations (CNVs) at the single‐cell level were inferred using the *inferCNV* R package (version 1.4.0; https://github.com/broadinstitute/inferCNV/wiki). Normal luminal epithelial cells from Henry et al. [29] were incorporated as reference controls to distinguish malignant from diploid profiles. Analyses were performed with stringent parameters, including the “denoise” option, a hidden Markov model (HMM) for CNV state prediction, and a cutoff threshold of 0.1 to minimize false‐positive calls.

To enhance the accuracy of CNV detection, we further applied a Bayesian latent mixture model, which enabled probabilistic refinement of true CNV events. For each cell, the mean CNV signal was estimated across all 22 autosomes, generating a genome‐wide view of chromosomal alterations. To standardize comparisons across the dataset, gene expression values were normalized to a scale of −1 to 1 prior to integration with CNV profiles. Finally, a CNV instability score was derived by computing the quadratic sum across CNV regions, providing a quantitative measure of genomic instability at the single‐cell resolution.

### 4.10 Cell Cluster Scoring

Differentially expressed genes (DEGs) within epithelial compartments were identified using a threshold of logarithmic fold change (logFC) greater than 2 and *p* < 0.05. Gene expression matrices were curated from the Oncomine database to ensure consistency across cohorts. To quantify epithelial transcriptional activity, we derived an epithelial score for each sample, defined as the aggregate expression of all identified DEGs, thereby capturing a composite measure of epithelial gene expression.

Samples were subsequently stratified into three clinically relevant categories: normal prostate tissue, localized PCa, and metastatic lesions. Pairwise comparisons were performed using the Wilcoxon signed‐rank test, while multi‐group comparisons were assessed using the Kruskal–Wallis H test. A significance threshold of *p* < 0.05 was applied throughout, designating statistically meaningful transcriptional differences across tissue states.

### 4.11 Cell–Cell Communication Network Analysis

To dissect intercellular signaling within the TME, we applied the Cellchat framework (http://www.cellchat.org/) [55]. After standard preprocessing of expression matrices, the “secreted signaling” database was used as the reference to model ligand–receptor interactions. Overexpressed genes and interactions were identified using the functions identifyOverExpressedGenes, identifyOverExpressedInteractions, and projectData with default parameters, thereby enabling systematic prioritization of biologically relevant communication signals.

Communication probabilities between cell populations were inferred using the computeCommunProb, computeCommunProbPathway, and aggregateNet functions, which collectively allowed pathway‐level aggregation of signaling events. The resulting interaction networks were visualized as heatmaps and hierarchical graphs, delineating both the intensity and topological hierarchy of signaling relationships across distinct cell types.

To highlight key mediators of intercellular crosstalk, ligand–receptor pairs were further visualized using bubble plots, providing an intuitive display of dominant signaling pathways. Together, this analysis generated a comprehensive network view of cell–cell communication, enabling insights into the molecular architecture of epithelial–stromal–immune interactions in prostate cancer progression.

### 4.12 Trajectory Analysis of Fibroblasts

To reconstruct the developmental dynamics of fibroblasts, we employed Monocle2 (http://cole‐trapnell‐lab.github.io/monocle‐release/docs_mobile/). Genes expressed in at least 10 cells were retained to ensure robust statistical robustness and minimize noise from low‐abundance transcripts. DEGs with a *q*‐value < 0.01 across cell clusters were used to establish the ordering of cells, thereby defining the transcriptional continuum underlying fibroblast states.

Trajectory inference was performed using the reduceDimension function with the DDRTree algorithm, followed by orderCells to assign pseudotime values to individual cells. This framework enabled the mapping of fibroblast progression along a pseudotemporal axis, capturing dynamic transcriptional transitions. Expression dynamics of lineage‐defining marker genes were visualized with the plot_cell_trajectory function, providing high‐resolution insights into state‐specific regulatory changes during fibroblast differentiation.

To further dissect transcriptional plasticity, DEGs along pseudotime were identified by setting the fullModelFormulaStr parameter to “Pseudotime,” which allowed precise modeling of gene expression as a function of cellular progression. This approach facilitated the characterization of stage‐specific transcriptional programs and highlighted candidate regulators of fibroblast state transitions within the TME.

### 4.13 Cell Culture

The human prostate cancer cell lines DU145 (#TCHu222), PC3 (#CRL‐1435), LNCaP (#CRL‐1740) and 22Rv1 (#TCHu100), as well as the human embryonic kidney cell line 293T (#CRL‐11268), were obtained from the American Type Culture Collection (ATCC, Manassas, VA, USA)., which had been authenticated by short tandem repeat (STR) fingerprinting. To ensure culture fidelity, all cell lines were routinely authenticated and tested for mycoplasma contamination using PCR‐based assays. PC3, LNCaP and 22Rv1 cells were maintained in RPMI‐1640 medium (Gibco), whereas DU145 and 293T cells were cultured in Dulbecco's modified Eagle's medium (DMEM; Gibco). Both media were supplemented with 10% fetal bovine serum (FBS; Gibco) and 1% penicillin–streptomycin (Meilun) to provide essential nutrients and prevent microbial contamination. C4‐2B cells were continuously cultured in the presence of enzalutamide for six months to establish the enzalutamide‐resistant C4‐2R cell line. The NE1.3 cell line was kindly provided by Prof. Jun Qin from the Shanghai Institute of Nutrition and Health. All cells were maintained at 37°C in a humidified incubator with 5% CO_2_. All cell lines were routinely tested for mycoplasma contamination, and the results were consistently negative.

### 4.14 Lentiviral Constructs, Production, and Infection

Short hairpin RNAs (shRNAs) targeting *TSPAN1* and *NRXN1* were designed using the Thermo Fisher Scientific RNAi design tool (https://rnaidesigner.thermofisher.com/). Oligonucleotide pairs containing AgeI‐ and EcoRI‐compatible overhangs (Table S2) were synthesized (Tsingke Biotechnology), annealed, and cloned into the pLKO.1 lentiviral backbone.

Lentivirus was generated using a third‐generation packaging system. Briefly, lentiviral transfer constructs were co‐transfected with pVSVG, pRSV‐rev, and pMDLg/pRRE helper plasmids into 293T cells using polyethylenimine (PEI; DNA:PEI ratio of 1:3) in Opti‐MEM (Thermo Fisher Scientific). Following a 20‐min incubation at room temperature, the transfection mixture was applied to cells. After 24 h, the medium was replaced with fresh growth medium, and viral supernatants were collected at 48 and 72 h post‐transfection. Supernatants were clarified by filtration through 0.45 µm filters to remove cellular debris.

For transduction, target cells at ∼70% confluency in six‐well plates were incubated with a mixture of viral supernatant, fresh medium (1:1), and polybrene (10 µg/mL) to enhance infection efficiency. After 36 h, cells were subjected to puromycin selection until non‐transduced cells were completely eliminated. Surviving populations were expanded, stabilized, and subsequently assessed for knockdown efficiency by RT–qPCR analysis of target gene expression.

### 4.15 siRNA Transfection

Small interfering RNAs (siRNAs) targeting NRXN1 (sense: 5′‐GCUGUUGUAAAGGAGAAACAA‐3′; antisense: 5′‐UUGUUUCUCCUUUACAACAGC‐3′) and a non‐targeting control siRNA (sense: 5′‐UUCUCCGAACGUGUCACGU‐3′; antisense: 5′‐ACGUGACACGUUCGGAGAA‐3′) were synthesized by Sangon Biotech (Shanghai, China) (Table S3). LNCaP cells were seeded at a density of 3 × 10^5^ cells per well in six‐well plates and transfected the following day with either 50 nM NRXN1 siRNA or control siRNA using Lipofectamine RNAiMAX transfection reagent (Thermo Fisher Scientific, USA), according to the manufacturer's protocol. Cells were harvested 72 h post‐transfection, and knockdown efficiency was validated by RT–qPCR.

### 4.16 Quantitative RT‐PCR

Total RNA was isolated using the EZ‐10 DNA Away RNA Mini Preps Kit (Sangon, China). For cDNA synthesis, 1 µg of purified RNA was reverse transcribed with the HiScript III RT SuperMix for qPCR (Vazyme, China). Quantitative RT–PCR (qRT–PCR) was performed on a LightCycler 480 system (Roche, Switzerland) using ChamQ Universal SYBR qPCR Master Mix (Vazyme, China) according to the manufacturer's protocol. Primer sequences are provided in Table S4.

Each experimental condition was analyzed in triplicate biological replicates, and experiments were independently repeated three times to ensure reproducibility. Threshold cycle (Ct) values were normalized to the housekeeping gene *GAPDH*, and relative gene expression levels were calculated using the 2^–ΔΔCt method.

### 4.17 Cell Viability and Proliferation Assays

Cells were harvested with trypsin and seeded into 96‐well plates at a density of 1 × 10^4^ cells/mL for DU145 and PC3 cells, and 4 × 10^4^ cells/mL for 22Rv1 cells. Cell viability and proliferation of DU145 and PC3 were assessed using the Cell Counting Kit‐8 (CCK‐8; Meilun, China), whereas 22Rv1 proliferation was evaluated using the MTT assay (Beyotime, China), following the manufacturers’ instructions.

Each experimental group was analyzed in quadruplicate at each time point, and assays were repeated independently to ensure reproducibility. Absorbance values were recorded using a microplate reader. Relative cell viability/proliferation was calculated accordingly. Statistical comparisons between groups were performed using two‐tailed Student's *t*‐tests, with *p* < 0.05 considered significant.

### 4.18 Colony Formation Assay

Cells were harvested with trypsin and seeded into six‐well plates at a density of 1 × 10^3^ cells per well for DU145 and PC3, and 4 × 10^3^ cells per well for 22Rv1. Following incubation, DU145 and PC3 cells were fixed with 4% paraformaldehyde and stained with crystal violet (Sangon, China) after 1 week, whereas 22Rv1 cells were processed after 2 weeks to allow colony maturation.

Colonies were imaged and quantified for each condition, with three biological replicates per group. Differences in clonogenic capacity between experimental groups were evaluated using two‐tailed Student's *t*‐tests, with *p* < 0.05 considered statistically significant. This assay provided a quantitative measure of long‐term proliferative and clonogenic potential under the specified experimental conditions.

### 4.19 Cell Migration Assay

Cells were harvested with trypsin and resuspended in serum‐free medium at a concentration of 2 × 10^5^ cells/mL. A 200 µL aliquot of the suspension was seeded into the upper chamber of 8 µm pore size transwell inserts (BD Biosciences, USA). The lower chambers were filled with 600 µL of RPMI‐1640 medium containing 20% FBS to act as a chemoattractant. After 48 h of incubation, cells were fixed with 4% paraformaldehyde and stained with crystal violet (Sangon, China). Non‐migrated cells on the upper surface of the membrane were removed with a cotton swab.

Migrated cells adherent to the underside of the membrane were imaged and quantified in three randomly selected microscopic fields per insert. Each condition was performed in triplicate. Statistical comparisons between groups were conducted using two‐tailed Student's *t*‐tests, with *p* < 0.05 considered significant. This assay provided a quantitative assessment of migratory capacity under defined experimental conditions.

### 4.20 Macrophage Isolation and In Vitro Polarization

Bone marrow‐derived macrophages (BMDMs) were generated as previously described [56]. Briefly, bone marrow cells were isolated from the femurs and tibiae of 8‐week‐old mice by flushing with sterile PBS. Red blood cells were removed using red blood cell lysis buffer for 5 min at room temperature, followed by two washes with cold PBS. The remaining nucleated cells were resuspended in BMDM complete medium consisting of high‐glucose DMEM supplemented with 10% FBS, 2 mM L‐glutamine, 100 U/mL penicillin, and 0.1 mg/mL streptomycin, and viable cells were counted prior to seeding.

For macrophage differentiation, cells were plated in 6‐well plates at a density of 1 × 10^6^ cells per well and cultured for 7 days, with medium replacement every 3 days. Following differentiation, cells were subjected to polarization under defined cytokine conditions. M1 polarization was induced using IFN‐γ (20 ng/mL) in combination with LPS (100 ng/mL), whereas M2 polarization was achieved using IL‐4 (20 ng/mL) and IL‐13 (20 ng/mL). To investigate the role of complement component C3 in macrophage polarization, recombinant C3 protein (MCE, HY‐P7863) was added to the designated experimental groups during the polarization phase.

After 48 h of stimulation, macrophages were harvested for RNA extraction, and RT–qPCR was performed to assess polarization‐associated gene expression. M1 polarization was validated by increased expression of Il6, Nos2 (iNOS), and Tnf, whereas M2 polarization was characterized by elevated expression of Arg1, Mgl2, Mrc1 (CD206), and Cd163. In addition, expression of genes associated with complement C3 receptor signaling was examined to evaluate pathway activation.

To extend these findings to a human system, THP‐1 monocytes were employed to establish an in vitro macrophage model. THP‐1 cells were seeded in 6‐well plates and stimulated with PMA (100 ng/ml) in complete medium for 48 h to induce differentiation into macrophage‐like cells. Successful differentiation was confirmed by morphological changes, including the transition from suspension growth to adherence and acquisition of a rounded, refractive morphology.

Polarization of THP‐1–derived macrophages was subsequently induced using IFN‐γ (50 ng/mL) plus LPS (100 ng/mL) for M1 conditions, or IL‐4 (20 ng/mL) plus IL‐13 (20 ng/mL) for M2 conditions. Recombinant human C3 protein (MCE, HY‐P7862) was added to the corresponding experimental groups to assess its regulatory effects on macrophage polarization in the human context.

To further investigate the functional consequences of C3‐driven macrophage polarization, M2‐polarized macrophages were co‐cultured with C3‐overexpressing WYPM‐1 cells. After 48 h of co‐culture, conditioned media were collected and applied to prostate cancer cell lines PC3 and DU145. Tumor cell proliferation was subsequently assessed to determine the impact of macrophage‐derived factors on cancer cell growth.

### 4.21 Sphere Formation Assay

For three‐dimensional sphere formation, 22Rv1 cells were seeded at a density of 300 cells per well in the center of 24‐well plates precoated with 50 µL of a 1:1 mixture of Matrigel (BD Biosciences, USA) and Advanced DMEM/F12 medium (Gibco, USA) supplemented with B27 (Invitrogen, USA), 20 ng/mL EGF, and 10 ng/mL bFGF. After allowing the cells to settle for 1 h, 500 µL of complete sphere culture medium was added to each well, and the medium was replenished every 2 days.

Sphere formation and morphology were monitored by phase‐contrast microscopy on days 7, 9, and 11. The total number of spheres per well was quantified, and sphere‐forming efficiency was calculated to assess the self‐renewal and tumor‐initiating capacity of 22Rv1 cells under defined conditions.

### 4.22 3D Matrigel Clone Formation Assay

For three‐dimensional clonogenic growth, 22Rv1 cells were resuspended in Advanced DMEM/F12 medium supplemented with B27 (Invitrogen, USA), 20 ng/mL EGF, 10 ng/mL bFGF, and 5% Matrigel (BD Biosciences, USA). A total of 500 cells per well were seeded into ultra‐low‐attachment 24‐well plates to prevent adherence and promote clonal sphere formation. Cultures were maintained with medium replenishment every 2 days.

After 9 days, clonal efficiency was evaluated by quantifying spheres with diameters > 50 µm under a 20× objective microscope. Both the percentage of effective clones and the mean sphere area were calculated to assess clonogenic potential. Statistical comparisons between groups were performed using unpaired two‐tailed Student's *t*‐tests, with *p* < 0.05 considered significant. This assay provided a quantitative measure of self‐renewal and anchorage‐independent growth capacity of 22Rv1 cells under three‐dimensional conditions.

### 4.23 In Vivo Xenograft Mouse Model

Six‐week‐old male BALB/c nude mice were purchased from GemPharmatech (Jiangsu, China) and maintained under specific pathogen‐free conditions with free access to food and water. DU145 and PC3 cells were harvested, washed twice with PBS, and resuspended in serum‐free medium at a concentration of 1 × 10^8^ cells/mL. For implantation, 50 µL of the cell suspension was mixed 1:1 with Matrigel (Yeasen, China) and subcutaneously injected into the right flank of each mouse.

Tumor growth was monitored weekly by caliper measurement, and volumes were calculated using the formula V = (L × W^2^)/2, where L represents tumor length and W represents tumor width (in millimeters). After 4 weeks, mice were euthanized, and tumors were excised, measured, and weighed to evaluate tumor burden. This xenograft system enabled in vivo assessment of prostate cancer cell growth dynamics and provided a platform to investigate tumor biology under physiologically relevant conditions.

### 4.24 Construction of Neuroendocrine‐Type Castration‐Resistant Prostate Cancer (CRPC‐NE) Cell Lines

LNCaP cells were maintained at 37°C in a humidified incubator with 5% CO_2_ in RPMI‐1640 medium (ATCC, USA) supplemented with 10% fetal bovine serum (FBS; Hyclone, USA) and 1% penicillin–streptomycin. To induce a neuroendocrine (NE) phenotype, cells were cultured for 15 days in phenol red–free RPMI‐1640 medium (Gibco, USA) supplemented with 5% charcoal‐stripped FBS (CS‐FBS; Newzerum, China) and 1% penicillin–streptomycin. This hormone‐depleted culture condition facilitated the differentiation of LNCaP cells toward a neuroendocrine‐like state.

### 4.25 Immunostaining and Immunofluorescence

Formalin‐fixed, paraffin‐embedded (FFPE) prostate tissue specimens were sectioned at a thickness of 4 µm. Tissue sections were first deparaffinized in xylene and rehydrated through a graded ethanol series. Antigen retrieval was subsequently performed by heating the slides in 10 mM sodium citrate buffer (pH 6.0) at 98°C for 15 min to unmask epitopes.

Following antigen retrieval, sections were processed for either immunohistochemical (IHC) or immunofluorescence (IF) analysis as described below.

For IHC analysis, slides were processed using the PV‐9000 detection kit (Origene, China) according to the manufacturer's instructions. Immunoreactivity was visualized using 3,3′‐diaminobenzidine (DAB; Origene, China) as the chromogenic substrate. Stained sections were counterstained (if applicable), dehydrated, and mounted prior to imaging. Brightfield images were captured using an Olympus photomicroscope (Olympus, Japan).

For IF staining, slides were washed three times with DPBS (5 min each) after antigen retrieval and then blocked with 5% donkey serum for 40 min at room temperature to reduce nonspecific binding. Sections were incubated with primary antibodies overnight at 4°C. After three washes in DPBS, slides were incubated with species‐appropriate fluorophore‐conjugated secondary antibodies. Nuclei were counterstained with DAPI. Fluorescent images were acquired using a TCS SP8 confocal laser‐scanning microscope (Leica, Germany).

The following primary antibodies were used: anti‐NRXN1 (Sigma, Cat. ABN161‐I, 1:1000), anti‐DCN (Abcam, Cat. ab189364, 1:100), anti‐C3 (Sigma, Cat. HPA020432, 1:1000), anti‐APOD (Sigma, Cat. HPA040520, 1:500), anti‐PTGDS (R&D Systems, Cat. MAB10099, 1:60), anti‐CXCL1 (Abcam, Cat. ab89318, 1:100), anti‐CD163 (Abcam, Cat. ab156769, 1:100), anti‐α‐SMA (Invitrogen, Cat. MA1‐06110, 1:100), anti‐STEAP4 (Proteintech, Cat. 11944‐1‐AP, 1:500), anti‐MYH11 (Abcam, Cat. ab133567, 1:250), and anti‐PD‐1 (Cell Signaling Technology, Cat. 86163T, 1:300).

Secondary antibodies included the following Alexa Fluor–conjugated antibodies (all from Invitrogen, used at 1:500 dilution): Donkey anti‐Mouse IgG (H+L), highly cross‐adsorbed (Cat. A‐21202, Alexa Fluor 488); Donkey anti‐Rabbit IgG (H+L), highly cross‐adsorbed (Cat. A‐31572, Alexa Fluor 555); Donkey anti‐Goat IgG (H+L), cross‐adsorbed (Cat. A‐11055, Alexa Fluor 488); and Donkey anti‐Rabbit IgG (H+L), highly cross‐adsorbed (Cat. A‐31573, Alexa Fluor 647). Detailed antibody information is provided in Table S5.

### 4.26 Cell Immunofluorescence Staining

LNCaP cells were seeded on glass coverslips following transfection and fixed with 4% paraformaldehyde for 30 min at room temperature. Cells were permeabilized with 0.03% Triton X‐100 for 30 min and subsequently blocked with donkey serum for 40 min. Primary antibodies were applied overnight at 4°C, including rabbit anti‐NSE (Abcam, Cat.ab79757,1:200) and rabbit anti‐synaptophysin (Abcam, Cat.ab32127,1:500). After washing, cells were incubated with Alexa Fluor 555–conjugated anti‐rabbit secondary antibody (Invitrogen,Cat. A‐31572, 1:500) for 1.5 h at room temperature. Nuclei were counterstained with DAPI (1 µg/mL; Thermo Fisher Scientific, USA) for 7 min. Fluorescence images were captured using an Olympus photomicroscope (Olympus, Japan) under identical exposure settings.

### 4.27 Western Blot Analysis

Cell membrane proteins were isolated using the Membrane and Cytosol Protein Extraction Kit (EpiZyme) in accordance with the manufacturer's protocol. Protein concentrations were determined prior to electrophoresis to ensure equal loading. Equivalent amounts of protein were separated by SDS–PAGE on 8% or 12% polyacrylamide gels, selected according to the molecular weight of the target proteins, and subsequently transferred onto 0.45 or 0.2 µm polyvinylidene difluoride (PVDF) membranes (Merck Millipore, USA).

After transfer, membranes were blocked with 3% bovine serum albumin (BSA) for 1.5 h at room temperature to reduce nonspecific binding. The membranes were then incubated with primary antibodies overnight at 4°C. Following extensive washing with TBST to remove unbound antibodies, membranes were incubated with species‐appropriate secondary antibodies. Immunoreactive bands were detected using an enhanced chemiluminescence (ECL) system and visualized with the ImageQuant LAS 500 imaging platform (USA).

The following primary antibodies were used: anti‐CACNA1H (Proteintech, Cat. No. 28092‐1‐AP; 1:500), anti‐ORAI1 (Proteintech, Cat. No. 66223‐1‐Ig; 1:20,000), anti‐ORAI3 (Proteintech, Cat. No. 25766‐1‐AP; 1:300), anti‐TRPM8 (Sigma, Cat. No. SAB2104241; 1:1,000), and anti‐ATP1A1 (Proteintech, Cat. No. 14418‐1‐AP; 1:5,000) (Table S5).

### 4.28 Membrane Potential Assays

Cell membrane potential was quantified using the bis‐(1,3‐dibarbituric acid)‐trimethine oxanol (DiBAC4(3)) assay kit (Solarbio, China) according to the manufacturer's protocol. DiBAC4(3) is a voltage‐sensitive fluorescent dye that preferentially enters depolarized cells, thereby enabling quantitative assessment of membrane potential changes.

Briefly, cells were harvested by enzymatic dissociation, washed twice with Dulbecco's phosphate‐buffered saline (DPBS), and resuspended in complete Dulbecco's modified Eagle medium (DMEM) supplemented with 10% fetal bovine serum and 1% penicillin–streptomycin. The DiBAC4(3) dye was then added to the cell suspension at a final concentration of 1 µmol/L. Cells were incubated at 37°C in a humidified atmosphere containing 5% CO_2_ for 60 min to allow adequate dye uptake.

Following incubation, cells were washed again with DPBS to remove excess extracellular dye and minimize background fluorescence. Fluorescence intensity, which reflects alterations in membrane potential, was subsequently measured by flow cytometry (BD C6 Plus, USA) and visualized by fluorescence microscopy (Olympus, Japan). Flow cytometry data were analyzed using FlowJo software (version 10.8.1).

### 4.29 Cytosolic Calcium Ion Detection

Intracellular cytosolic Ca^2^^+^ levels were determined using the fluorescent calcium indicator Fluo‐4 AM (Servicebio, China) in accordance with the manufacturer's protocol. Fluo‐4 AM is a membrane‐permeable acetoxymethyl ester that, upon intracellular de‐esterification, emits fluorescence proportional to cytosolic Ca^2^^+^ concentration.

Briefly, cells were washed twice with Dulbecco's phosphate‐buffered saline (DPBS) to remove residual culture medium and serum components. Cells were then incubated with Fluo‐4 AM working solution (Fluo‐4 AM diluted 1:500 in calcium ion detection buffer) at 37°C for 30 min in a humidified incubator to allow probe loading.

Following incubation, the staining solution was removed, and cells were washed twice with DPBS to eliminate excess extracellular dye. Cells were subsequently resuspended in calcium ion detection buffer and incubated for an additional 15 min at 37°C to ensure complete intracellular de‐esterification of the probe, thereby enabling stable fluorescence signal generation.

Finally, Ca^2^^+^‐associated fluorescence intensity was quantified by flow cytometry (BD Accuri C6 Plus, USA) and visualized using fluorescence microscopy (Olympus, Japan). Flow cytometric data were processed and analyzed using FlowJo software (version 10.8.1).

### 4.30 Published Datasets Used in this Study

To comprehensively delineate the transcriptional dynamics underlying prostate cancer (PCa) progression and treatment‐induced remodeling, we systematically integrated multiple publicly available transcriptomic datasets encompassing spatial transcriptomics, bulk RNA sequencing (RNA‐seq), and single‐cell RNA sequencing (scRNA‐seq) platforms. This multi‐layered design enabled cross‐validation of findings across disease stages, therapeutic contexts, and cellular resolutions.

To characterize disease evolution across clinical stages, we analyzed GSE278936, which provides consecutive spatial transcriptomic profiling spanning benign prostatic hyperplasia (BPH), primary PCa, ADT‐treated PCa, castration‐resistant prostate cancer (CRPC), and metastatic PCa (MET) [45]. To complement these spatial data, GSE183100 and GSE150368 were incorporated to assess transcriptional alterations in bulk prostate tissues following ADT, thereby enabling systematic evaluation of hormone‐induced remodeling at the tissue level.

To resolve cellular heterogeneity associated with ADT response, we examined HRA006365, a scRNA‐seq dataset profiling PCa tissues before and after ADT [57, 58]. This dataset facilitated cell‐type–specific analysis of adaptive transcriptional programs. In parallel, multiple independent bulk RNA‐seq cohorts, including GSE6099 [59], GSE6919 [60], GSE10645 [61], GSE3325 [62], GSE70768 [63], GSE80609 [64], GSE6752 [65], GSE6959 [66] and GSE74367 [67], were integrated to compare gene expression profiles across normal adjacent benign prostate tissue, primary PCa, and metastatic PCa, thereby strengthening stage‐dependent validation.

To model castration resistance in vitro, we analyzed datasets focused on hormone‐resistant progression in PCa cell lines, including GSE95413 [68], GSE56829, GSE147541 [69], GSE151083 [70], and GSE150368 [71]. These datasets enabled characterization of transcriptional adaptations accompanying androgen‐independent growth and long‐term androgen deprivation.

Given the clinical relevance of NEPC, we further incorporated datasets comparing adenocarcinoma and NEPC phenotypes, including GSE104786 [72], GSE33054 [73], GSE55945 [74], GSE3933 [75], as well as the Singh [76], Welsh [77], Liu [78], Vanaja [79], and LaTulippe [80] cohorts. These resources enabled systematic interrogation of gene expression signatures associated with neuroendocrine differentiation and lineage plasticity.

To identify molecular drivers linked to castration resistance, GSE32269 [81] was analyzed to compare hormone‐dependent PCa with metastatic CRPC. Additionally, GSE29650 [82], comprising CRPC patient tissues, was used to validate correlations among KLK3, TSPAN1, and the AMACR^+^ epithelial signature.

To further interrogate neuroendocrine lineage reprogramming, we analyzed GSE154576, GSE118206 [83], and GSE59984 [84], which compare gene expression profiles between neuroendocrine PCa cell lines or tissues and conventional adenocarcinoma cell models, including 22Rv1, LNCaP, DU145, and VCaP.

To validate the functional relevance of *NRXN1* in NEPC, we incorporated data from a genetically engineered mouse model with dual *TP53* and *RB1* loss [85], as well as clinical and genomic cohorts including GSE35988 [51], SU2C_Cell 2015 [53], NatMed 2016 [52], and TCGA_Cell 2015 [54]. Associations between *NRXN1* expression and NEPC‐related features were further examined using TCGA data obtained from FireBrowse.

Finally, to assess androgen‐dependent regulation of *NRXN1*, we analyzed GSE8702 [86], which profiles longitudinal progression of LNCaP cells toward androgen independence and demonstrates progressive upregulation of *NRXN1* during androgen deprivation. In addition, GSE71797 [87] was used to evaluate transcriptional responses to androgen (R1881) stimulation in 22Rv1 and VCaP cells.

Collectively, the integration of these complementary datasets enabled a multidimensional and stage‐resolved evaluation of transcriptional reprogramming across PCa initiation, androgen deprivation, castration resistance, and neuroendocrine differentiation, thereby ensuring robust mechanistic inference and cross‐cohort validation.

## Author Contributions

Y.C., J.C., Z.M., and G.‐H.W. conceived and designed the study. Y.C., Q.Z., and M.W. developed methodologies and software. Formal analyses were performed by Y.C., D.D., J.L., N.L., M.L., Q.Z., J.C., G.‐H.W., and Z.M., with validation by D.D., M.L., and G.‐H.W. Experimental investigations were carried out by Y.C., D.D., J.L., N.L., M.L., Q.Z., S.C., Q.L., T.L., T.L., C.Z., Q.S., Q.W., H.L., J.X., C.L., M.W., J.C., G.‐H.W., and Z.M. Resources were provided by Y.C., C.Z., M.W., J.C., Z.M., and G.‐H.W. Data curation and visualization were led by Y.C. with contributions from D.D. and others. Supervision and project administration were provided by Y.C., J.C., Z.M., and G.‐H.W. Funding was secured by Y.C., J.C., Z.M., and G.‐H.W. Y.C. and G.‐H.W. drafted the manuscript, and all authors contributed to review and editing.

## Conflicts of Interest

The authors declare no conflicts of interest.

## Supporting information




**Supporting File**: advs76594‐sup‐0001‐SuppMat.xls.

## Data Availability

The data that support the findings of this study are available from the corresponding author upon reasonable request.
